# NOD-like receptors in fish: evolution, structure, immune signaling, and targeting for aquaculture vaccine adjuvants

**DOI:** 10.3389/fimmu.2025.1665071

**Published:** 2025-09-02

**Authors:** Banikalyan Swain, Kavi R. Miryala

**Affiliations:** Department of Infectious Diseases & Immunology, College of Veterinary Medicine, University of Florida, Gainesville, FL, United States

**Keywords:** NOD-like receptors (NLRs), teleost fish immunity, pattern recognition receptors (PRRs), inflammasome signaling pathway, aquaculture vaccine development, host-pathogen interactions in fish

## Abstract

Teleost fish possess a highly diverse innate immune system, which is well-adapted to the pathogen-rich aquatic environment in which they reside. NOD-like receptors (NLRs), a conserved family of cytosolic pattern recognition receptors, are at the center of this defense mechanism, activating immune responses, recognizing pathogen-associated molecular patterns (PAMPs) and damage-associated molecular patterns (DAMPs). Here, we present an integrative overview of the current state of fish NLRs in terms of their evolutionary diversification, structural framework, signaling pathways, and functional roles in the context of bacterial, viral, and parasitic pathogens. We discuss six principal NLRs: nucleotide-binding oligomerization domain-containing protein 1 (NOD1), NOD2, NLRC3, NLRC5, NLR family member X1 (NLRX1), and NLR family pyrin domain-containing 1 (NLRP1), highlighting their domain structures, 3D conformations, and downstream signal chains. We focused on the immune regulatory roles of NLR family acidic transactivation domain-containing (NLRA) and NLR family CARD domain-containing (NLRC) subfamily components, the formation of the NLRP1 inflammasome, and the new roles of mitochondrial-specific NLRs in antiviral immunity. We discuss future directions for NLRs as immunological targets in aquaculture, referencing known NLR-activating adjuvants, exploring their ligand specificity, and highlighting challenges like functional redundancy. Much of the insight into the fish NLRs in this review comes from their well-researched mammalian counterparts. NLR-based immune modulation represents the ability of these receptors to detect microbial or danger signals and regulate key signaling pathways, such as nuclear factor kappa-light-chain-enhancer of activated B cells (NF-κB), activator protein 1 (AP-1), interferon regulatory factors (IRFs), and inflammasome activation. These pathways help shape the immune response by negatively or positively altering cytokine production and improving antigen presentation. By bringing together what we know about NLR evolution, structure, and function, this review aims to support new ideas and research into how fish defend themselves from disease and how we might strengthen that defense through improved vaccine and adjuvant design.

## 1 Introduction

Fish in aquatic environments constantly face challenges that trigger innate immunity, such as the introduction of pathogens and stressors. Within this first line of defense, components of pattern recognition receptors (PRRs) trigger downstream signaling cascades that lead to inflammation, cytokine production, apoptosis, and pathogen clearance (1). This occurs in the presence of pathogen-associated molecular patterns (PAMPs) and damage-associated molecular patterns (DAMPs), such as flagellin, nucleic acids, lipopolysaccharides (LPS), γ-D-glutamyl-meso-diaminopimelic acid (iE-DAP), lipoproteins, muramyl dipeptide (MDP), glucans, N-formylmethionine, and toxins (2, 3). The sources of these elements originate from bacterial, viral, and parasitic infections that activate various PRRs in fish, including Toll-like receptors (TLRs), the most heavily investigated, as well as retinoic acid-inducible gene-I-like receptors (RLRs), C-type lectin receptors (CLRs), and the subject of this review, NOD-like receptors (NLRs) (4).

NLRs are emerging as key sensors in pattern-triggered immunity (PTI) and are located intracellularly in the cytoplasm, unlike the majority of TLRs, which are located either on the cell surface or within intracellular compartments. Depending on the subfamily of the protein, NLRs act as positive or negative regulators of pro-inflammatory mediated responses through nuclear factor kappa-light-chain-enhancer of activated B cells (NF-κB) and activator protein-1 (AP-1) via mitogen-activated protein kinase (MAPK) signaling, and interferon regulatory factors (IRFs) (3, 5, 6). Upon activation, NF-κB is a transcription factor that activates pro-inflammatory and cell survival genes, producing cytokines (7–9). At the same time, MAPK is a highly conserved family of serine/threonine kinases that transmits PAMPs/DAMPs from the cell membrane to the nucleus through a phosphorylation cascade, ultimately producing AP-1, which regulates immune responses, apoptosis, development, and stress adaptation in fish (5, 10). In addition, specific subfamilies of NLRs are activated as inflammasomes via pro-caspase-1 signaling, making them modulators and negative regulators of innate immunity to maintain a stable immune response through various pathways (2, 3, 5, 11).

NLRs consist of an N-terminal effector domain, a central nucleotide-binding oligomerization domain (NBD), and a C-terminal leucine-rich repeat (LRR) domain, forming a tripartite structural arrangement (3, 12, 13). Each of these three domains plays a distinct role, which is vital within the PTI. The effector domain participates in signal transduction through protein interactions (13). This results in the distinction of five key subfamilies of NLR proteins: NLR family acidic transactivation domain-containing (NLRA), NLR family baculoviral inhibitor of apoptosis repeat (BIR)-containing (NLRB), NLR family caspase activation and recruitment domain-containing (NLRC), NLR family pyrin domain-containing (NLRP), and NLR family proteins with alternative effector domains (NLRX) (3, 12, 14, 15). Further distinctions across these classes will be detailed in section 2.1. NBD is responsible for oligomerization mediation and contains ATPases associated with diverse cellular activity (AAA+) subdomains for ATPase activity (3, 16, 17). This is also known as the NACHT domain, whose name originates from the four proteins that it was first identified in, NAIP, CIITA, HET-E, and TP1 (17). Along with this, the C-terminal segment, consisting of the LRR domain, plays a vital role in recognizing PAMPs/DAMPs upon entering the cell by binding to the ligands present (13). These domains are present in most NLRs, with evolutionary variations leading to exceptions (18). Further functional domains may also be present as a result of evolutionary adaptations across various taxa or factors of divergence within NLR subfamilies. Given this, NLRs are structurally conserved across all known vertebrates, with fish displaying a unique expansion, structurally and functionally, most likely shaped by pathogen-driven selective pressures (18, 19). Unlike vertebrates, non-vertebrates lack an adaptive immune system, resulting in an evolutionary expansion of PRRs, such as in corals and sea urchins (18, 20). NLRs were initially discovered in mammalian species and later began to be researched in plants, reptiles, amphibians, birds, and most importantly, fish — the taxonomic class that will be expanded on in this review.

The role of fish NLRs in disease resistance is of increasing interest, especially in aquaculture, where infectious diseases threaten global fish production. Upon activation following ligand binding, NLRs can act as innate immunity pathogen receptors and regulate MHC gene expression depending on their classification (13). Along with this, some NLRs display inflammasome function, boosting cytokine release, antigen presentation, and adaptive immunity, allowing for their implementation in vaccine development as targets for adjuvant strategies (21).

In this review, we focus on six key members of the NLR family: NOD1, NOD2, NLRC3, NLRC5, NLRX1, and NLRP1. We base our selection on four main criteria: (i) their presence in North American and well-studied fish, (ii) the availability of full-length, annotated amino acid sequences in public databases like NCBI and Ensembl, and (iii) the existing or emerging functional evidence from studies on gene expression, domain structure, or immune responses. (iv) frequency of co-study between NLRs presented in this review. While NLRP3 has been studied extensively in cyprinids such as zebrafish (10.1074/jbc.RA119.011751) and grass carp (https://doi.org/10.1016/j.fsi.2024.109367), many available sequences are partial or not verified in these species. Also, NLRP3 fails criterion iv, as it is independently identified in experimental assays of current literature. On the other hand, NLRP1, though less functionally studied, is found in multiple genomes with complete sequences, which allows for comparative analysis.

This review explores the historical overview of NLR discovery, evolutionary classification, domain structure, and 3D modeling analysis (generated by AlphaFold), signaling mechanisms, functional roles in various pathogen defenses, and implications for vaccine or adjuvant development in aquaculture. The functional basis of these proteins offers key future directions that can be applied in other aspects of veterinary medicine.

## 2 Nomenclature, history, evolution of NLRs

The NLR family is an evolutionarily conserved family of PRRs located intracellularly (22). Identification of this class of proteins began in mammals in the 1990s as a key player in the activation of innate immune responses via inflammation, host defense, and autoimmune diseases. Despite sharing common roles in immunity, the classification, evolutionary patterns, and structural features in mammals and teleosts differ.

**Figure 1:**
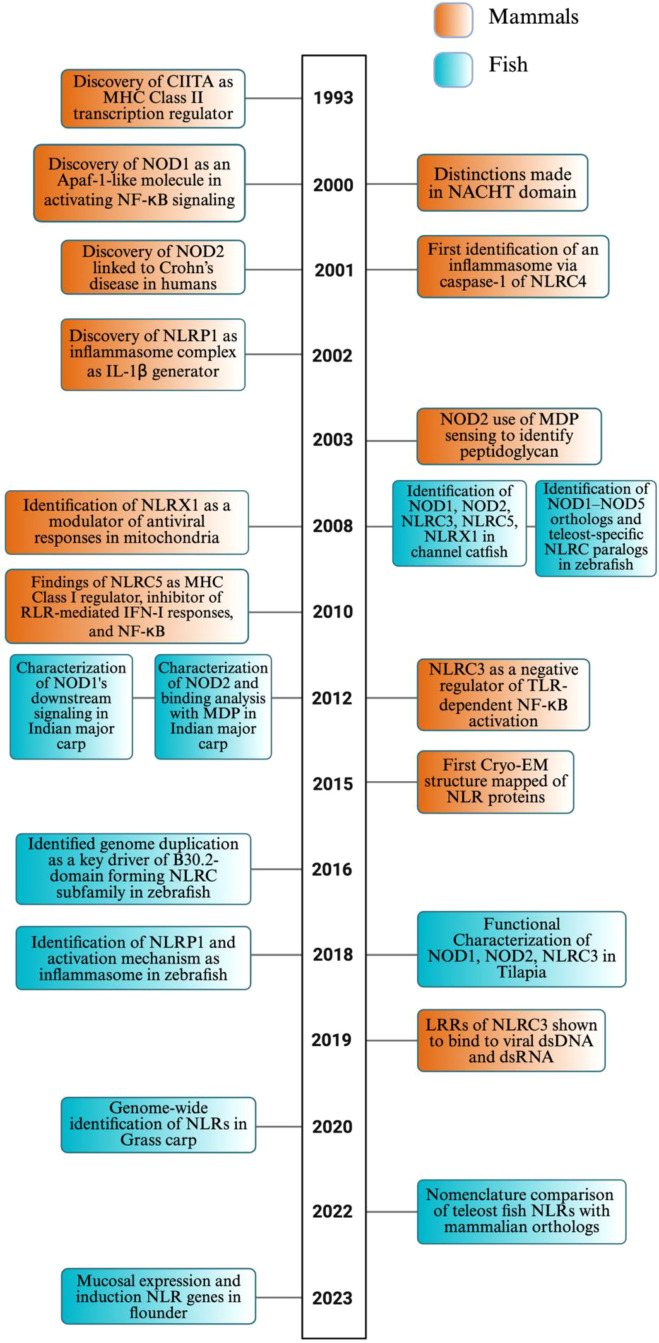
Timeline of NLR discovery and evolution in mammals and fish. This timeline highlights pivotal discoveries and major conceptual advances in the study of NOD-like receptors (NLRs) in both mammals (orange) and fish (teal), spanning from the early characterization of mammalian NLR functions in immunity to the growing body of research focused on teleost-specific genes, functions, and evolutionary mechanisms. The timeline underscores the increasing contribution of fish studies in recent years, revealing both conserved and lineage-specific insights into NLR biology. While this timeline captures several defining milestones in NLR research, we recognize that it does not include many additional contributions to the field due to space constraints. The image was created using BioRender.com.

This section will discuss the diversity of nomenclature systems used for NLRs across species, highlighting the inconsistency in teleost classification and the requirement for a uniform nomenclature based on phylogenetic relationships. We will then outline the historical timeline of key milestones in NLR discovery from early mammalian research to the more specialized work in fish (**Figure 1**). Following this, the evolutionary diversification of NLRs in teleosts and mammals will be considered, drawing on phylogenetic analyses that reveal conserved patterns and species-specific adaptations. Lastly, the functional domain elements will be detailed, followed by 2D and 3D structural characterizations. Together, these topics provide a foundation for understanding the diversity and rationale of NLRs across piscine species for subsequent sections on the functional investigation in immune signaling.

### 2.1 Nomenclature

The discovery of a majority of NLRs was initially done in mammals due to their accessibility and significance to human health. This resulted in delayed research in fish that have displayed a unique characterization of NLRs, which varies in gene expansion, domain architecture, and evolutionary lineage. Due to this complexity, a majority of fish species have been referred to with nomenclature that varies from the standardized nomenclature system established by the HUGO Gene Nomenclature Committee (HGNC) (23–25).

As mentioned, variations in the N-terminal segment constitute the distinction between these five subfamilies. In mammals, NLRA consists of an acidic activation domain, NLRB includes a baculovirus inhibitor of apoptosis repeat (BIR) domain, NLRC includes a caspase activation and recruitment domain (CARD), and NLRP has a pyrin domain (PYD) (3, 12, 14, 24). The NLRX subfamily varies, however, as it lacks an effector domain with a mitochondrial targeting sequence (MTS) near its N-terminal segment (26). In contrast, teleost fish exhibit a much more complex NLR repertoire, leading to distinct naming schemes. For example, zebrafish NLRs are grouped into NLR-A (NOD-like), NLR-B (NALP and NLRP-like), and the expansive, teleost-specific NLR-C subfamily, which includes hundreds of genes, many containing a C-terminal B30.2/PRY-SPRY domain (25, 27, 28). The B30.2 subfamily, also found in grass carp and other teleosts, likely evolved from a NOD3-like ancestor and does not exist in mammals. Likewise, grass carp categorize some NLRs as NLR-B30.2 as well (24). In contrast to these teleost-specific naming systems, species like channel catfish adopt a mostly mammalian-style nomenclature, using names such as NOD1, NOD2, NLRC3, NLRC5, and NLRX1, reflecting their structural similarity and simplifying cross-species comparison (24). Given this, even channel catfish have NLR proteins that are novel to teleosts (24, 27, 29). However, despite the shared names, proteins like “NLRC3” and “NLRC5” in zebrafish and grass carp may not be direct orthologs to their mammalian counterparts. This underscoresxthe broader issue: NLR nomenclature varies significantly between fish and mammals, and even among fish species due to species-specific gene duplication, structural divergence, and varying domain compositions, complicating orthology and functional inference.

Chuphal et al. provide a comprehensive naming comparison of these NLRs in several teleost fish to their mammalian orthologs (27). The teleosts mentioned in the study include: zebrafish, channel catfish, common carp, goldfish, Japanese flounder, olive flounder, rohu, grass carp, miiuy croaker, mrigal, orange-spotted grouper, Nile tilapia, catla, Atlantic salmon, rainbow trout, Asian seabass, point snout bream, turbot, Japanese pufferfish, spotted snakehead, and Ya-fish (27).

**Figure 2:**
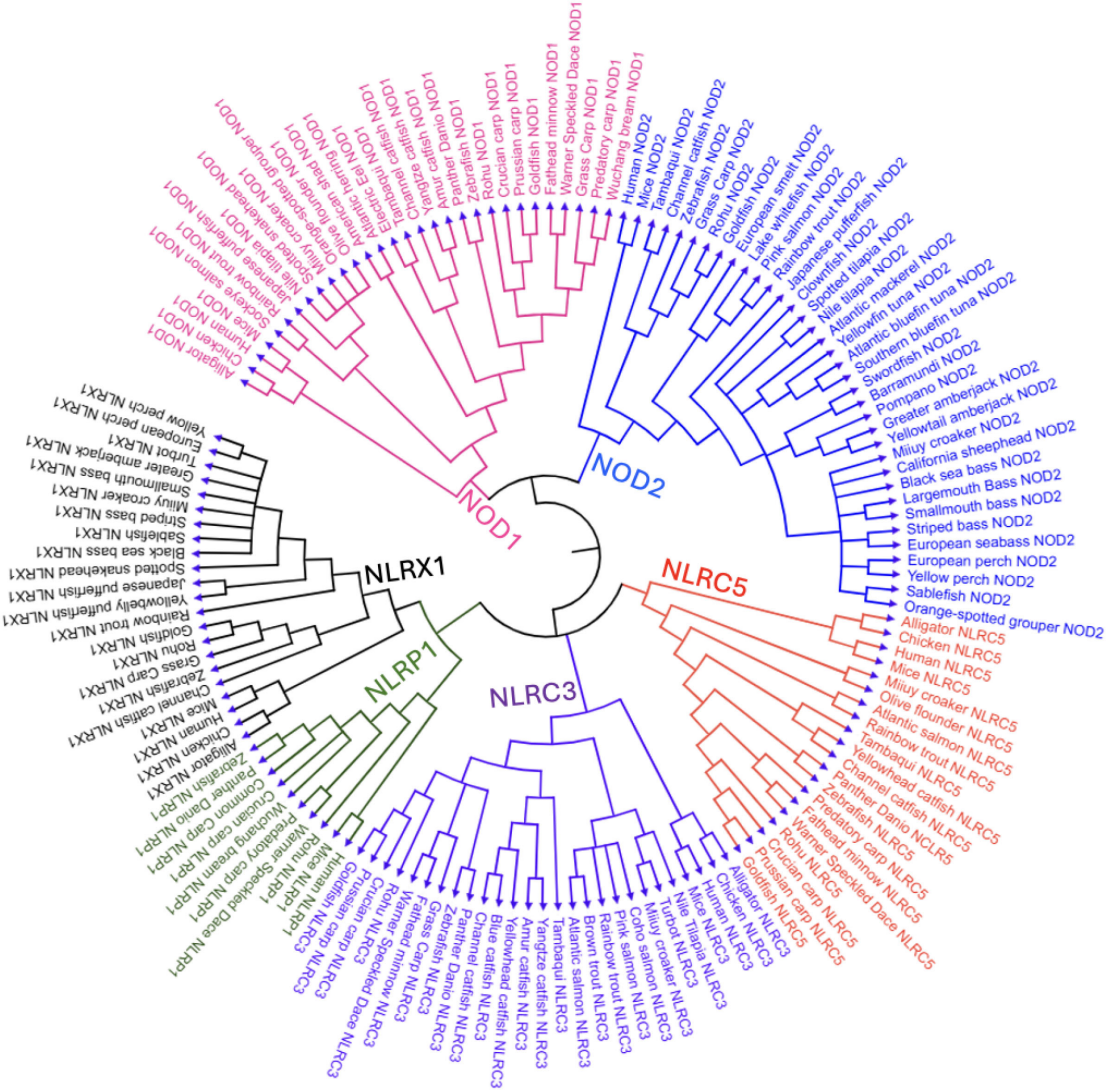
Phylogenetic tree of NLR proteins across vertebrate species, focusing on teleost fish.This circular phylogenetic tree shows the evolutionary relationships of various NLR subtypes—NOD1, NOD2, NLRC3, NLRC5, NLRX1, and NLRP1—from vertebrates such as teleost fish, amphibians (hourglass tree frog), reptiles (American alligator), birds (chicken), and mammals (mouse and human). Clades are color-coded: pink (NOD1), blue (NOD2), purple (NLRC3), orange (NLRC5), green (NLRP1), and black (NLRX1). We conducted a phylogenetic analysis of 57 freshwater and marine teleost fish—selected for their status as well-studied model organisms, evolutionary conservation across lineages, broad recognition, or representation of North American fauna—and five higher vertebrates, revealing both conserved and divergent patterns of NLR evolution within and across species. The phylogenetic tree was constructed using complete, full-length protein sequences of NLRs obtained from the NCBI’s protein database (GenPept). BLAST searches were conducted to identify proteins in other species containing >90% query coverage and an E value of 0 (30–33). FASTA sequences were aligned and analyzed using MEGA12 software, applying the neighbor-joining method to infer evolutionary relationships (34, 35).

**Figure 3:**
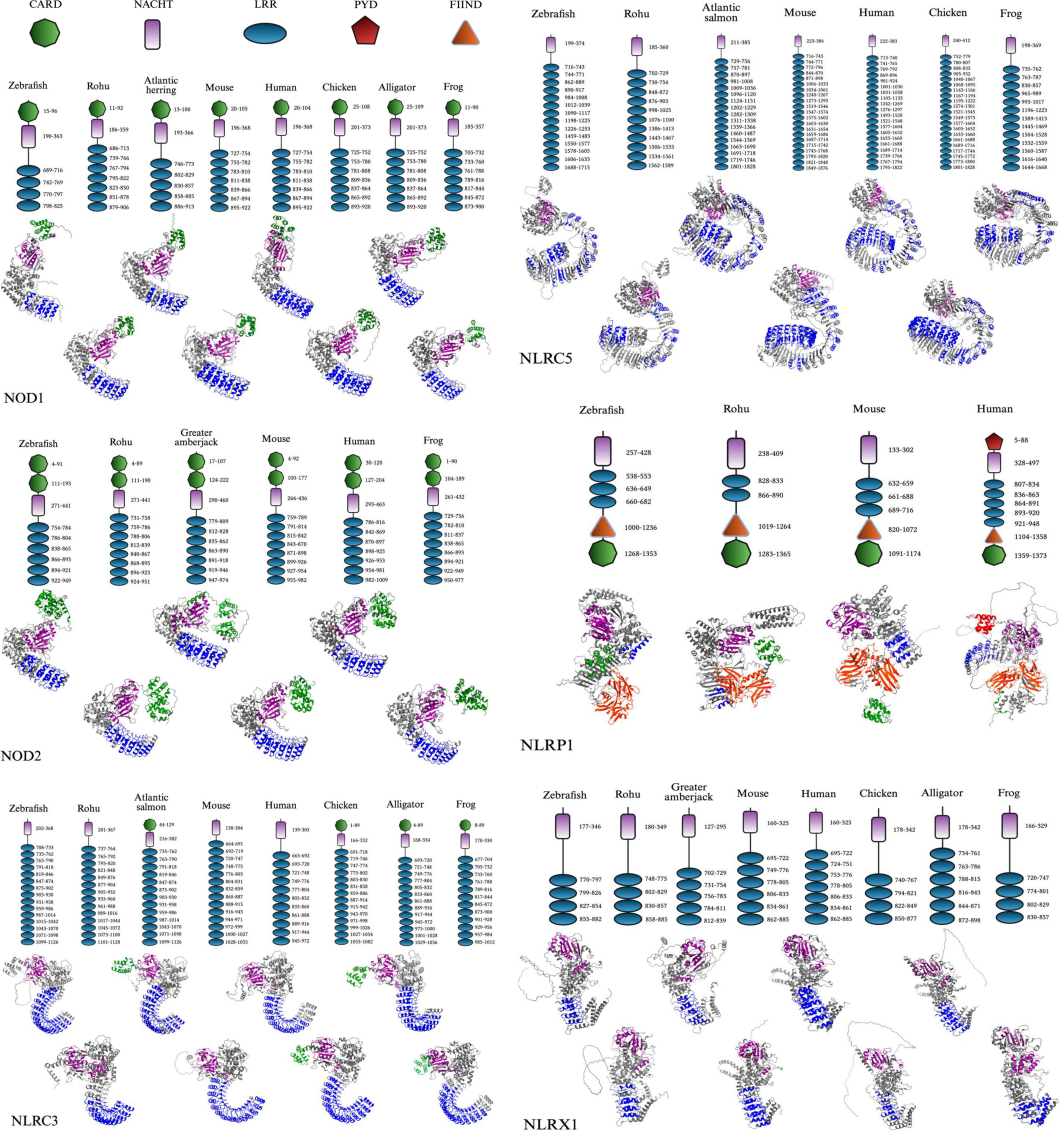
Comparative domain architecture and 3D structures of vertebrate NLR proteins. This figure displays the domain organization (top panels) and AlphaFold-predicted 3D structures (bottom panels) of six NOD-like receptors (NLRs): NOD1, NOD2, NLRC3, NLRC5, NLRX1, and NLRP1, across a diverse set of vertebrates including fish (e.g., zebrafish, rohu, Atlantic salmon, Atlantic herring, and greater amberjack) and representative tetrapods (e.g., mouse, human, chicken, American alligator, and Hourglass tree frog). Each protein’s domain architecture includes the CARD (green), NACHT (purple), LRR (blue), PYD (red), and FIIND (orange) domains, with the domain key shown in the final panel. The same structural domain legend also applies to all pathway figures referencing these proteins. FASTA sequences were obtained from the NCBI protein database, and domain predictions were made using the SMART (Simple Modular Architecture Research Tool) database (36). 3D structures were generated using AlphaFold, and final visualizations were created with BioRender.com (37, 38).

In this review, we standardize the naming of NOD1, NOD2, NLRC3, NLRC5, NLRX1, and NLRP1 for all species for clarity. Although subfamily classification is historically based on the presence of specific N-terminal domains (e.g., CARD in NLRCs, PYD in NLRPs), evolutionary divergence has resulted in the loss or modification of these domains in some orthologs, particularly across vertebrate lineages. Furthermore, we do not introduce new nomenclature but rather adhere to standardized protein names provided by NCBI and Ensembl, along with recent literature conventions that correlated to the homology and clade of the protein leading to evolutionary conservation, rather than strict structural criteria as represented in the phylogenetic tree (**Figure 2**) and structural representations (**Figure 3**). For example, genes such as NLRC3 or NLRC5 may retain their designation despite lacking characteristic domains.

### 2.2 Discovery of NLRs in mammals

The majority of experimentation to characterize vital NLRs in mammals occurred in human and mouse species starting from the 1990s. **Figure 1** provides a condensed timeline of key mammalian discoveries, together with fish discoveries detailed in the following section, summarizing the findings discussed in the text. The founding member of the NLR protein family is CIITA, an MHC class II gene expression regulator, which was first discovered in 1993 in mice through complementation cloning (39). Its tripartite structure is similar to today’s known NLRs; however, the presence of a Proline, Serine, and Threonine (PST) rich domain located at the N-terminus contributes to functional differences as a transcriptional co-activator via nuclear localization and export signals in most antigen-presenting cells (APCs) (22, 40–43). This finding was followed by the discovery of nucleotide-binding oligomerization domain-containing protein 1 (NOD1) in mice as an Apoptotic protease activating factor-1 (Apaf-1)-like molecule containing CARD, nucleotide-binding domain (NBD), and LRR domains, the basis of NLRs (44). Apaf-1 is a cytosolic adaptor protein consisting of WD40 repeats, which is similar to that of LRRs in NOD1 (44–46). NOD1 was also found to perform homophilic CARD–CARD interactions to activate NF-κB by binding to Receptor–Interacting Serine/Threonine–Protein Kinase 2 (RIP2 or RICK), a kinase containing a CARD domain (44, 46). This activation is driven by NOD1’s ability to self-associate, which enables the recruitment of RIP2 to the γ subunit of the IκB kinase (IKKγ). Along with this, the NBD region or NACHT domain was further distinguished as a central ATPase core characterized by nucleotide binding and hydrolysis (16, 47, 48). These structural features allow the NACHT domain to regulate conformational changes essential for NLR oligomerization and activation in the presence of PAMPs/DAMPs (16). The discovery of NOD2 in 2001 was especially important as a frameshift mutation in the tenth LRR of NOD2 was found to impair its responsiveness to bacterial lipopolysaccharides by inhibiting NF-κB and altering the region of linkage on chromosome 16, resulting in increased susceptibility to Crohn’s disease in humans (49–53). In 2003, this was expanded on when NOD2 was shown to detect muramyl dipeptide (MDP), a conserved component of bacterial peptidoglycan, gram-negative but primarily gram-positive bacteria, due to their abundance of peptidoglycan (54, 55). NOD1, however, was shown to only detect meso-diaminopimelic acid (meso-DAP) in gram-negative bacteria, activating NF-κB (56, 57).

In a further human study in 2001, NLRC4 (now known as Ipaf) was identified to directly interact with and activate procaspase-1 via CARD–CARD interactions, establishing it as a specific and direct activator of caspase-1 (58, 59). Truncation of NLRC4’s LRR domain led to constitutive activation of caspase–1–dependent apoptosis, marking the first functional identification of an inflammasome-like platform in human cells (58, 59). The discovery of NLRP1 added to the class of inflammasome complex proteins, involving Apoptosis-associated speck-like protein containing CARD (ASC) or Pycard, caspase-1, caspase-5, and NALP1, a pyrin-domain protein homologous to NODs (60–64). Here, the ASC/Pycard is essential to mediate the cleavage of pro-IL-1β into its active form through the activity of caspase-1, resulting in a pro-inflammatory response (60–63).

The following NLRs have been shown to act as negative regulators of innate immunity to maintain immune homeostasis and protect tissues from damage. In 2008, researchers identified NLRX1 as a noncanonical mitochondrial NLR that acts as a modulator rather than a receptor of PAMPs (65). The protein interacted with the mitochondrial antiviral signal protein (MAVS), also known as Cardif, VISA, and IPS-1, at the mitochondrial membrane, disrupting its association with RIG-I and MDA5 (RLRs), negatively regulating IFN-I transcription, interferon regulatory factor 3 (IRF3), NF-κB, and generating reactive oxygen species (ROS), resulting in a unique function from the previously mentioned NLRs (65–71). Two years following this study, NLRC5 was shown to result in similar inhibitions of antiviral signaling with varied mechanisms from NLRX1. NLRC5 interacts with the same cytosolic sensor RLRs, resulting in the upstream inhibition of MAVS, suppressing IFN-I responses. Along with this, NLRC5 performed inhibitory binding to IKKα and IKKβ, preventing NF-κB signaling (72–74).

Apart from antiviral signaling, in response to cytoplasmic protein degradation peptides (type of DAMP), NLRC5 promotes MHC class I expression by acting as a type II interferon gamma (IFN-γ)–inducible transcriptional regulator that binds to MHC class I gene promoters such as HLA-A, HLA-B, and HLA-C to present antigens for CD8^+^ T cell docking and apoptosis (75, 76). This expression is vital, as although CIITA can transactivate MHC class I genes, its function as such is not widespread, limiting its expression specifically to lymphocytes and APCs and primarily functioning as a novel MHC class II regulator (75, 77–82).

NLRC3 acts as a negative regulator of innate immunity by binding to TRAF6, preventing its K63-linked ubiquitination (7). This, along with the upstream signaling interference via MyD88, resulted in the suppression of NF-κB activation, similar to several other NLRs and TLR signaling (7, 83). Unlike NLRC5 and NLRX1, which suppress MAVS-mediated type I interferon (IFN-I) signaling, NLRC3 specifically dampens TLR-driven inflammation as a distinct but complementary immunomodulator (7). NLRC3 was shown to directly bind to the ligand of viral double-stranded DNA (dsDNA) and, to a lesser extent, double-stranded RNA (dsRNA) via its LRR domain (84). This interaction enhances NLRC3’s ATPase activity and triggers a conformational change that causes it to release a stimulator of interferon genes (STING) and TANK-binding kinase 1 (TBK1) (84, 85). Freed TBK1 phosphorylates IRF3, lifting its suppression of the IFN-I pathway (84). NLRC3 functions to suppress CD4^+^ T cell activation, proliferation, and cytokine production, which dampens Th1 and Th17 responses through inhibition of NF-κB and extracellular signal-regulated kinase (ERK) signaling pathways (86). In both viral (LCMV) and bacterial (*Mycobacterium tuberculosis*) infections, NLRC3 deficiency enhanced protective immunity by promoting robust CD4^+^ T cell responses, positioning NLRC3 as a key modulator of adaptive immunity to prevent possible autoimmunity and chronic inflammation (86, 87). The modulators mentioned act as an immune “brake” to prevent overreaction of immunity, despite promoting pathogen survival in some cases due to this suppression of protective immunity.

In 2015, the first cryo-EM structure of an activated NLR inflammasome was presented, revealing that a single ligand-activated NAIP2 molecule results in conformational changes that nucleate the polymerization of multiple NLRC4 molecules (59). This was shown to form a large oligomeric complex, resembling a wheel, that activates downstream caspases and initiates the inflammatory response (59, 88–91). Prior to cryo-EM’s structuring, X-ray crystallography was used to map the 3D structure of these proteins, but this method required crystallized proteins, which were often difficult to obtain, and lacked the ability to capture multiple conformational states (92). Protein mapping has advanced with tools like AlphaFold in 2018, which uses neural networks to predict 3D structures from amino acid sequences by leveraging evolutionary data and structural templates (37).

### 2.3 Discovery of NLRs in fish

With the rising economic value of aquaculture globally, immunological studies on NLRs in fish have gained traction, with the first classification occurring in 2008. In channel catfish, structural, phylogenetic, and genomic analyses characterized NOD1, NOD2, NLRC3, NLRC5, and NLRX1 (93). These PRRs were constitutively expressed in cell lines of leukocytes and other tissues in catfish with enteric septicemia caused by *Edwardsiella ictaluri* (93). With this, zebrafish orthologs for all five mammalian NOD proteins (NOD1–NOD5) were identified as well as over 200 teleost-specific NLRC genes, many encoding a B30.2 domain (25). Phylogenetic analysis showed that NLRC likely evolved from a NOD3-like ancestor, and RT-PCR confirmed expression of NLRA, NLRB, and NLRC genes in the intestine, liver, and spleen (25). The naming of these zebrafish NLRs varies from what will be used in this review, as detailed in section 2.1.

In 2012, the first characterization of downstream signaling by NOD1 in Indian major carp (rohu) was performed by Swain et al. The full-length NOD1 cDNA was mapped, and its domains were displayed (94). Exposure to LPS and poly I:C led to robust, tissue-specific upregulation of NOD1 (up to 80-fold in blood) and its adaptor RIP2, indicating activation of downstream NF-κB and MAPK pathways (94). Several other studies have used LPS and poly I:C to elicit varied NLR responses in fish species. Furthermore, pathogen challenge with *A. hydrophila*, *E. tarda*, and *S. flexneri* confirmed inducible NOD1–RIP2 signaling *in vivo* (94). Soon after, the first structural insights into ligand recognition by fish NOD1 identified LRR1–2, LRR3–7, and LRR8–9 as critical binding motifs for poly I:C, LPS, and γ-D-glutamyl-meso-diaminopimelic acid (iE-DAP), respectively, and confirmed conserved NOD1 signaling activation in rohu (95). This was performed through methods of molecular docking and 6-ns molecular dynamics simulation (95). Swain et al. also characterized NOD2 in rohu, and observed upregulation of the protein (~4-fold at 4 hours and ~7-fold at 24 hours) in peripheral blood leukocytes upon muramyl dipeptide (MDP) stimulation, confirming MDP as a potent ligand for rohu NOD2 (95). Much like NOD1, NOD2 displayed concurrent upregulation of the downstream adaptor protein RIP2 (~6.3-fold at 4 hours), establishing both NODs as key cytosolic sensors in teleost innate immunity (95, 96). In mrigal, similar RIP2 upregulation (~5.6-fold in 6 hours) was seen upon MDP stimulation *in vivo* along with upregulation of IL-1β (~6-fold in 6 hours), resulting in a pro-inflammatory response (96). Similar upregulation of these NODs was discovered in Nile tilapia, goldfish, miiuy croaker, Japanese flounder, and catfish (previously mentioned) upon ligand and pathogen introduction, possibly indicating conserved signaling pathways across teleost fish (93, 97–100).

In 2016, Howe et al. identified the origins of these teleost-specific NLRC genes in zebrafish, B30.2 domains that underwent extensive tandem and segmental duplications, especially on chromosome 4 (18). Similar genomic duplications and expansions were shown in other fish species, contributing to their naming discrepancies as mentioned. Following this, NOD1, NOD2, and NLRC3 were functionally characterized in Nile tilapia and shown to be upregulated at the mRNA and protein levels in response to *Streptococcus agalactiae*, with distinct tissue-specific expression patterns (97). They demonstrated that NOD1 activates NF-κB in a ligand-independent manner, whereas NOD2 requires MDP, and NLRC3 requires either MDP or iE-DAP to enhance NF-κB signaling (97). This upregulation of NF-κB signaling represents the context-dependent role as a regulator in fish, whereas in mammals, NLRC3 is known to inhibit the NF-κB pathway. Around this same time, the first activation mechanism of an inflammasome was identified in zebrafish as an NLRP1 homolog (DrNLRP1) (28). Here, two pro-inflammatory caspases, DrCaspase-A and DrCaspase-B, are activated in an ASC-dependent manner where homotypic interactions occur. Interestingly, the study was the first to identify the teleost-specific presence of PYD instead of CARD in DrCaspase-A/B, which was later replicated in channel catfish (28, 101). DrCaspase-A is activated first to initiate IL-1β processing, followed by DrCaspase-B to complete its maturation (28).

A genome-wide study in 2020 systematically identified 65 NLR genes in grass carp (24). These subfamilies, along with most other fish NLRs that have been characterized genomically, have been organized with their mammalian orthologs in 2022, bringing clarity to the field as mentioned in 2.1 (27). Recently, flounder NLR genes were highly expressed in mucosal tissues such as gills, skin, and hindgut following intraperitoneal and immersion vaccination with inactivated *Vibrio anguillarum* (13). Notably, the teleost-specific NLR-C subfamily members, especially those with B30.2 domains, showed the most pronounced immune responses, emphasizing their potential role in mucosal immunity (13).

Together, these findings display the earliest discoveries of evolutionary innovation and functional diversification of NLRs in teleosts, driven by gene duplication events and selective pressures from aquatic pathogens. As immunological tools and genomic resources continue to expand, fish NLRs, especially teleost members, are becoming increasingly valuable for guiding vaccine development in aquaculture.

### 2.4 Evolution and diversity of fish NLRs

A circular phylogenetic tree was generated, based on full-length protein sequences for six NLR subtypes (NOD1, NOD2, NLRC3, NLRC5, NLRX1, and NLRP1) across diverse vertebrate species, emphasizing teleost fish (**Figure 2**). The amino acid sequences used to construct this phylogenetic tree, along with their accession numbers, are provided in **Supplementary Table S1**. The teleost fish selected for this phylogenetic analysis were chosen for their commercial importance and representation across diverse aquatic environments, ranging from freshwater to saltwater habitats. The selection criteria used to generate the tree dictated which NLRs were included in this review; because NLRP3 failed to meet most of those criteria, it was omitted. Each NLR subtype formed separate clades, color-coded to show their evolutionary relationships. NOD1 (pink) and NOD2 (blue) are presented to be highly conserved across teleosts, forming sister groups that underscore their shared evolutionary origin. NOD2 is ubiquitous in fish species; however, it is not present in some of the presented vertebrates.

The NLRC3 and NLRC clades appeared across fish and non-fish species, forming closely related sister clades that suggest a common ancestral origin and divergence through gene duplication. NLRP1 (green clade) clustered compactly and basally with fewer teleost members, implying limited duplication and elements of conservation. It was more closely related to NLRX1 than to other NLRs, suggesting a deeper shared ancestor. Likewise, NOD1 and NOD2 formed a sister clade, highlighting duplication-driven diversification. Catfish, tuna, and bass each formed species-specific clades, indicating within-group evolutionary relationships, while NOD2 was uniquely observed in tuna species among the taxa represented.

Overall, the phylogenetic tree highlights a clear divergence between teleosts, likely in response to aquatic pathogen diversity and environmental pressures. In addition to the diverse representation of teleost fish, the phylogenetic tree also includes key higher vertebrates, such as mammals (human, mouse), birds (chicken), reptiles (alligator), and amphibians (hourglass tree frog), which serve as reference points for evolutionary comparison. These species consistently form distinct, well-supported clades from teleost branches within each NLR subtype. Given that teleosts represent an early-diverging lineage among vertebrates, the observed clade structure supports the inference that fish are ancestral to these higher vertebrate lineages. The driving force of evolutionary divergence in these non-aquatic vertebrate NLRs likely reflects adaptation to terrestrial environments, including exposure to novel pathogens and immune challenges.

### 2.5 Key functional domains of Fish NLRs

The CARD is a conserved protein–protein interaction module commonly found at the N-terminus of NLRs and was found on NOD1, NOD2, NLRC3, and NLRP1 in the proteins we mapped (**Figure 3**). The CARD domain is a part of the death domain (DD) superfamily, which also includes pyrin and death effector domains. Members of this superfamily typically adopt a six-helix bundle fold with a bent or disrupted H1 helix along with a hydrophobic core that stabilizes the structure (102–104). The functional interactions are generally driven by charge complementarity across helices H1–H4, often forming filament-like assemblies that serve as signaling platforms (105, 106). As a result, CARDs mediate homotypic interactions, enabling recruitment of adaptor proteins and downstream effectors involved in apoptotic and inflammatory signaling pathways, including caspase activation and NF-κB regulation (107–109). Although CARD sequences show low identity, their structural conservation allows for interaction diversity and pathway specificity (110, 111). Noncanonical variants like the untypical CARD (uCARD) domain of NLRC5 also exist, which have been shown to remain solvent-exposed in both open and closed states and contain a nuclear localization signal (NLS), implicating a potential dual role in both signaling and nuclear import (112).

The NACHT (NAIP, CIITA, HET-E, and TP1) domain belongs to the NB-ARC superfamily of signal-transducing modules and was found in all mapped NLRs (16, 104). Structurally, the NACHT domain consists of several subregions: NBD (Walker A and Walker B motifs), helical domain 1 (HD1), HD2, and winged-helix domain (WHD), which coordinate ATP binding and hydrolysis as mentioned (16, 47, 48, 113). These subdomains form a tightly packed core, with the NBD centrally located and flanked by regulatory helices and a winged-helix fold that stabilizes the domain structure and promotes conformational changes necessary for oligomerization and downstream signaling (102, 114, 115). The domain has since been identified to drive the assembly of multimeric complexes such as inflammasomes and apoptosomes in NLRs. Despite variability in adjacent domains, NACHT-mediated ATP binding is broadly required for the activation, autoinhibition release, and structural reorganization of these signaling platforms (16, 104).

LRR domains are found in all NLRs that were mapped across species, which emphasizes their functional necessity (**Figure 3**). LRRs are conserved motifs typically forming tandem arrays (two or more repeats), forming curved solenoid architecture ideal for protein-protein interactions (116, 117). This hook-type shape can be seen in the 3D display of the LRR region, colored blue (**Figure 3**). Each motif is typically around 20–30 amino acids and is characterized by a hydrophobic core and is made of an 11-residue pattern of LxxLxL where “L” is a hydrophobic amino acid such as leucine, isoleucine, valine, or phenylalanine, and “x” is any amino acid (118–121). This pattern supports a parallel β-sheet along the concave surface, represented as arrows on the 3D cartoon display indicating the polypeptide chain’s N-terminus to C-terminus direction (**Figure 3**) (118, 119). These β-sheets can only be seen in some angles, which were not completely displayed in all the NLRs in **Figure 3** to allow visualization of the other domains. However, the β-sheet surface is key for its ligand recognition properties, while other surfaces are also less commonly involved (116, 122–127). Also, this concave surface interacts with other domains, such as CARD, to maintain an inactive state, but this interaction is disrupted upon ligand binding, triggering activation through conformational changes (128, 129). With this, the truncation of the C-terminal LRR region often causes NLRs to become constitutively active as well (128, 130). The convex surface, however, is composed of α-helices, polyproline II helices, 3_10_ helices, β-turns, and β-strands that are interwoven (116, 118, 119, 131).

These three mentioned domains are the basis of NLRs, and at least two of these elements are present in every NLR that we mapped. The order of these domains consistently falls in the order of CARD, NACHT, and lastly the LRR region from the N- to C-terminus, except in NLRP1, which will be expanded on in the following section. Also, section 3.1 will display these domains’ binding affinities and their functional role in downstream signaling.

### 2.6 Structure and classification of fish NLRs

Understanding the structural organization of NLRs across diverse vertebrate species is critical for unraveling how innate immune recognition has evolved and diversified. Displaying both the 2D domain architecture and 3D protein conformation may offer insight into the conservation and divergence of key functional motifs such as CARD (caspase activation and recruitment domain), NACHT, LRR, transmembrane, coiled-coil, PYD (pyrin), and FIIND (function to find domain) (132). These regions of the protein collectively mediate pathogen recognition and signal transduction. By comparing representative species of teleost fish and other previously mentioned vertebrates, these structural visualizations represent shared evolutionary origins and lineage-specific adaptations that bridge sequence-based annotations with spatial protein dynamics, allowing for the interpretation of how structure relates to immune function across vertebrates. Some vertebrates and fish species lacked some of these NLRs, which is why there is no consistent display of the same species and a limited display of NLRP1.

The acidic activation domain, B30.2/PRY-SPRY domain, AAA+ domain, uCARD domain, and others could not be displayed in **Figure 3** as they did not reach the threshold to be mapped in the SMART database, despite possibly playing a role in immunogenicity (36). The AAA+ domain is especially important as it is a conserved subregion within the larger NACHT domain, responsible for ATP binding and oligomerization for inflammasome formation of NLRP1 following the cleavage within the FIIND domain, which will be discussed (115, 133). The nomenclature used in this paper is based on evolutionary history rather than the presence of specific domains, as not all proteins within a given subfamily consistently share the same domain architecture (**Figure 3**). Also, each NLR type maintains a relatively similar LRR range across species, though not often the same, while different NLR types display substantial variation (**Figure 3**). These patterns are likely driven by evolutionary pressures to recognize diverse PAMPs.

The 3D models reveal that the CARD domain appears to be surface-exposed and spatially distinct, supporting its role in initiating downstream signaling through homotypic interactions. For NOD1 and NOD2, this is especially important for binding to RIP2, while in NLRP1, ASC binding occurs to form the inflammasome. Likewise, the LRRs display a stereotypical bent architecture in the form of a curved solenoid projecting outward from the NACHT domain. Several of the mapped LRRs contain identical amino acid regions to other species mapped. These observations only apply to the applicable NLRs containing these domains. The NACHT domain, however, is present in every mapped NLR and forms the structural and functional core that connects upstream sensing to downstream effector activation through ATP-driven conformational changes. The AAA+ domain that controls this was not displayed in **Figure 3**, but is present in most NACHT domains displayed.

Several NLR-specific observations were made from the species in **Figure 3**. NOD1 displays a conserved domain organization across all examined vertebrate species, consisting of a single N-terminal CARD domain, a central NACHT domain, and a C-terminal array of LRR motifs. Regarding the 3D structure, functional studies have shown that NOD1’s CARD domain must present a negatively charged surface to effectively engage in the RIP2-CARD formation (3). It can also be observed that several of the displayed species have seven LRRs for iE-DAP and other ligand binding. NOD2 contains tandem CARD domains, which are conserved across both teleost and non-teleost species, indicating functional importance. Also, the number of LRRs ranged from six to eight, which likely preserves its function to recognize MDP. This consistent structural organization of domains can only be seen in these two NLRs, suggesting that selective pressures have maintained the signaling framework while permitting mild interspecies variation in domain repeat number and sequence.

NLRC3 exhibits more variability at the N-terminus, where only some species—including Atlantic salmon, chicken, alligator, and frog, retain a CARD domain. However, in NLRC5 and NLRX1, no CARD domains are present. This suggests an evolutionary gain or loss of the CARD domain, with its presence in some teleosts indicating functional divergence. The NLRC subfamily also seems to display the first instance of a considerably expanded LRR region, often over ten repeats, with even more in NLRC5, possibly indicating a varied capacity for ligand discrimination. NLRC5 is unique as the LRR domains have notable discontinuities in the 3D models, appearing as structural gaps, contributing to a much longer amino acid sequence with possible flexible loop regions within the LRR segment. The protein’s structural features likely enable its role in controlling nuclear trafficking, where histone acetyltransferase-mediated retention in the nucleus enhances its ability to activate MHC class I expression (134). Much like NOD1, NLRX1 consists of four to seven LRRs, with a straighter solenoid structure.

NLRP1 shows notable divergence from other NLRs in both domain architecture and species distribution. Structurally, NLRP1 is distinct in 3D conformation, with a more compact, globular fold and a unique arrangement of domains involved in inflammasome activation. In humans, NLRP1 contains an N-terminal PYD, which differs from the C-terminal CARD domain displayed in fish species of rohu and zebrafish. This is also unique, as the other NLR subfamilies have a CARD at the N-terminus. Also, all mapped NLRP1 proteins contain a conserved FIIND domain, along with NACHT and LRR domains. The functional role of these domains will be further detailed in section 3.3. The order of these domains is as follows: PYD (if present), NACHT, LRR, Function to Find Domain (FIIND), and CARD. Despite slight structural variability, domain conservation in these NLRs allows for the preservation of function in microbial sensing, inflammasome formation, and pro-inflammatory effects throughout vertebrate evolution.

## 3 NLR signaling pathways in fish

This section details how NOD1, NOD2, NLRC3, NLRC5, NLRX1, and NLRP1 in fish activate immune responses through several key signaling cascades. NOD1 and NOD2 recognize bacterial peptidoglycan fragments, iE-DAP and MDP, respectively, triggering the recruitment of RIP2 and subsequent activation of inflammation. NOD1, NOD2, NLRC5, and in some teleosts, NLRC3 display characteristics of adaptive immune response through the upregulation of IFN-Is, cytokines, and MHC-I transcription, which will be detailed.

NLRC3 and NLRC5 are recognized as negative regulators of immune signaling, acting to constrain excessive inflammation, and at times can promote pathogen survival. Moreover, NLRC3 was shown to act as a positive regulator at times, specifically in some teleosts. While both belong to the NLR family, they engage in distinct inhibitory interactions with signaling hubs involved in innate immunity, particularly those tied to IFN-1 and cytokine expression, along with MHC Class I promotion.

NLRX1 localizes to mitochondria and negatively regulates antiviral signaling by disrupting STING and MAVS interactions, thereby reducing IFN-I responses, as well as negative regulation of cytokines in a similar mechanism to NLRC3. Meanwhile, NLRP1 varies by contributing to inflammasome formation by interacting with ASC and caspase proteins. These proteins are structurally different in piscine species, resulting in varied homotypic interactions, which will be expanded on.

Several other pathways may also be present, but will not be displayed in the signaling pathway figures due to their relative rarity and space constraints. Currently, research on parasite-induced NLR expression has been lacking, which is why it is not shown with the bacterial and viral pathogenic components in the following signaling pathways. However, it should be noted that NLRs are vital in resistance to parasitic infection in teleosts.

### 3.1 NOD1 and NOD2

**Figure 4:**
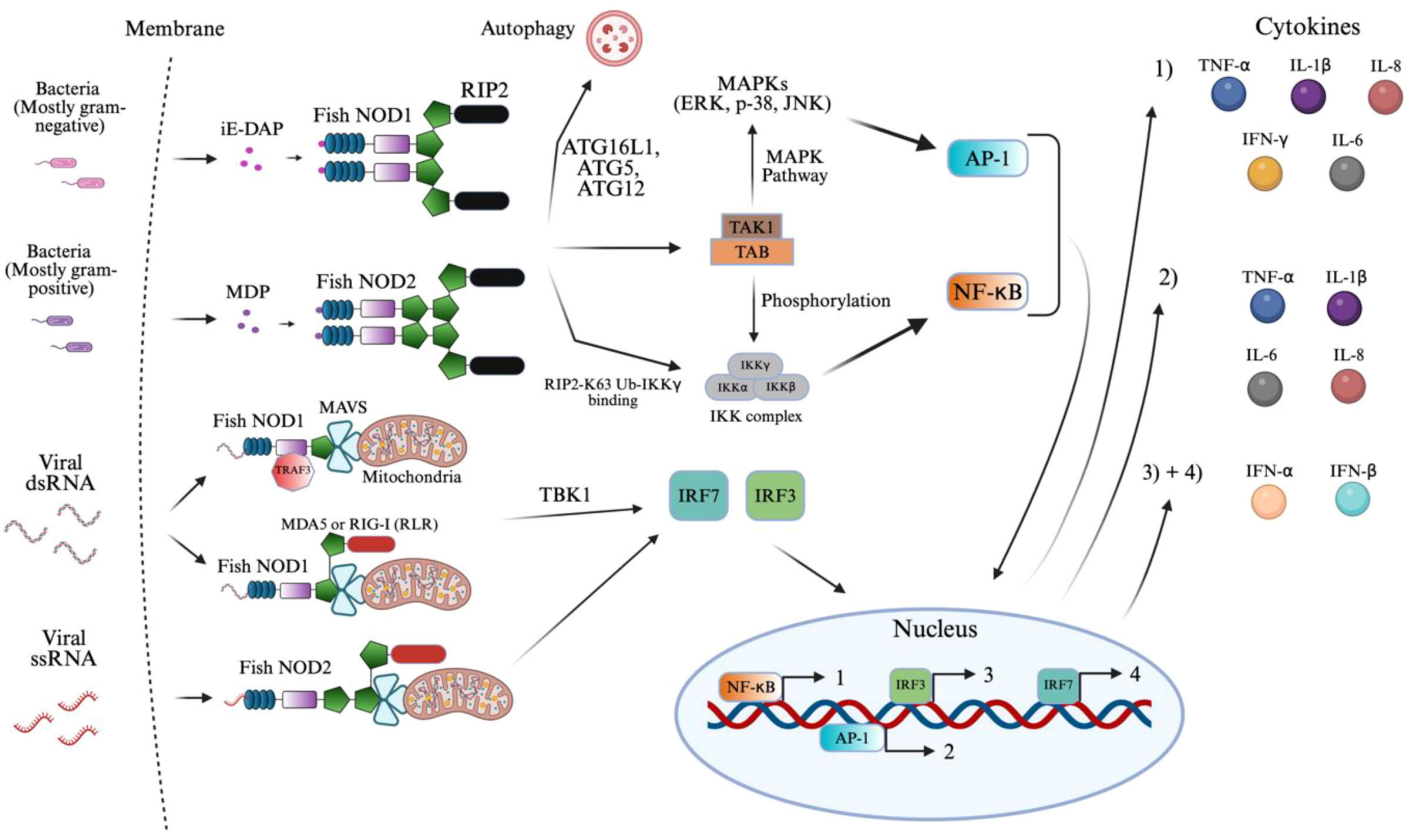
NOD1 and NOD2 signaling activation of innate immunity. The figure illustrates the signaling pathways activated by NOD1 and NOD2 in response to bacterial and viral ligands in teleost fish. NOD1 and NOD2 recognize distinct bacterial elements, such as iE-DAP and MDP, respectively, along with viral elements of dsRNA and single-stranded RNA (ssRNA), respectively, in teleost fish. Ligand binding activates RIP2, binding to NOD1/2 that recruits TAK1 and initiates AP-1 and NF-κB signaling. NOD2 also interacts with MAVS mediated via MDA5, RIG-I, and TRAF3 to activate TBK1 and IRF pathways during viral detection. The resulting transcriptional responses lead to the production of pro-inflammatory cytokines (1–2) and interferons (3–4), as indicated by the color-coded circles. The image was created using BioRender.com.

**Figure 4** summarizes the established signaling pathways of NOD1 and NOD2 in fish, which are described in detail below. NOD1’s LRR domain binds to iE-DAP, a PAMP released by gram-negative and, to a lesser extent, gram-positive bacteria and acid-fast bacteria such as Mycobacterium (56). This binding triggers a conformational change that allows NOD1 to recruit RIP2 (135–137). Similar to iE-DAP, MDP is released from the peptidoglycan of primarily gram-positive bacteria, due to its abundance, which is recognized by NOD2, similarly recruiting RIP2 (54, 135–137). It should be noted that gram-negative bacteria also release MDP, but in trace amounts, resulting in variability of NOD2 signaling (54). The RIP2 signaling complex is formed when two NOD1/NOD2 molecules dimerize upon ligand binding and recruit two RIP2 adaptors through CARD–CARD interactions, creating a stable 2:2 NOD1–RIP2 tetrameric signaling unit (135–137). This initiates K63-linked polyubiquitination, in which ubiquitin molecules are sequentially attached via their lysine-63 residues to form a flexible, linear chain that serves as a scaffold for recruiting signaling proteins rather than marking substrates for degradation by K48-linked chains (138). This structure and binding were not visually displayed in **Figure 4** to avoid confusion with other signaling pathways. These ubiquitin chains then serve as docking sites for IKKγ (NEMO), by binding to its ubiquitin-binding domains (138). This interaction facilitates the spatial proximity required for the TAK1-TAB kinase complex to phosphorylate and activate the catalytic subunits IKKα and IKKβ within the IKK complex (138–140). Once activated, the IKK complex phosphorylates the cytoplasmic heterodimer of IκB proteins, leading to their degradation and the release of NF-κB (138, 141, 142). Freed from its inhibitor, NF-κB translocates into the nucleus, where it binds to consensus DNA-binding κB sites, located in the promoter regions of target genes (141). This promotes the transcription of genes encoding key pro-inflammatory cytokines such as TNF-α, IFN-γ, IL-6, IL-1β, and IL-8, resulting in a robust innate immune response (96, 139, 143–145). NF-κB was also shown to modulate *ntl* expression, the zebrafish ortholog of the mammalian Brachyury gene, which is involved in mesoderm formation and notochord development during early embryogenesis in zebrafish (141). This represents influences in developmental gene expression. In parallel, TAK1 also activates MAPKs, driving AP-1-mediated transcription and releasing TNF-α, IL-1β, IL-6, and IL-8 (96, 144–148). The exact MAPKs found across teleost fish are unclear; however, one study in ayu (*Plecoglossus altivelis*) classified the phosphorylation of ERK, p38, and JNK through western blotting analysis, which may represent conserved MAPKs across other teleost fish (144).

The antiviral signaling pathway is kicked off through the binding of viral dsRNA or ssRNA (149–152). Here, cytosolic NOD1/2 collaborate with MDA5, a RIG-like receptor (RLR) through homotypic CARD interactions (149, 153). The RNA binding itself occurs in the helicase/CTD regions of MDA5, resulting in oligomerization that exposes its CARDs, docking onto MAVS and NOD1/2 (149, 153–157). Retinoic acid-inducible gene (RIG-I) in place of MDA5 has also been shown to display similar interactions in Zebrafish (149, 153–156). Much like these RLRs, an adaptor protein, TNF Receptor–Associated Factor 3 (TRAF3) recruits MAVS on NOD1 specifically (149, 156, 158). Following the formation of the MAVS complex, mediated by these MAVS recruiting proteins, TBK1 activates to phosphorylate interferon regulatory factors, IRF3 and IRF7, leading to their dimerization and translocation into the nucleus (154, 158). The result is a key driver in innate immunity through the formation of IFN-Is (IFN-α/β) as well as IFN-stimulated genes (ISGs) (149, 153–156, 158). This integrated signaling network results in a vast transcriptional program coordinated by NF-κB, AP-1, IRF3, and IRF7 to counter both bacterial and viral infections. The proteins have not been found to detect viral DNA, suggesting their ligand specificity is biased toward RNA.

NOD1/2 have a clear role in innate immunity and the subsequent priming of adaptive immune components. Although not represented in **Figure 4**, the production of IFNα/β has been found to activate dendritic cell maturation, increasing antigen presentation (159, 160). Similarly, TNF-α, IL-1β, IL-6, and IFN-γ increase MHC and costimulatory molecule expression on antigen-presenting cells. These features support Th1 differentiation and create an inflammatory environment that improves CD4^+^ and CD8^+^ T cell priming (159–161).

A study on Grass carp (*Ctenopharyngodon idella*) demonstrated that NOD1 recruited autophagy-related genes to initiate autophagosome formation (162, 163). These autophagosomes subsequently fuse with lysosomes, enabling the lysosomal degradation of the gram-negative bacteria, highlighting a NOD1-dependent autophagy pathway in the presence of iE-DAP in teleost fish (162, 164). Autophagy is a highly conserved catabolic process where these cytoplasmic components, including intracellular pathogens, are sequestered into double-membraned autophagosomes formed through the conjugation activity of autophagy-related proteins such as ATG16L1, ATG5, and ATG12 (162–164). This signaling pathway is similar in mammals as well (164).

Due to the complexity of **Figure 4** and space constraints, the signaling pathway of ROS (reactive oxygen species) expression by NOD2 was not displayed. Following the previously mentioned NOD2 binding with MDP, the plasma membrane translocates, where it physically interacts with the dual oxidase (DUOX2) via its LRR domain (165). This interaction, aided by the DUOXA2 activator, stimulates DUOX2 to produce ROS, specifically hydrogen peroxide (165, 166). The resulting ROS contributes directly to bacterial killing and amplifies NOD2-mediated NF-κB signaling and cytokine production (165–167).

### 3.2 NLRC3 and NLRC5

**Figure 5:**
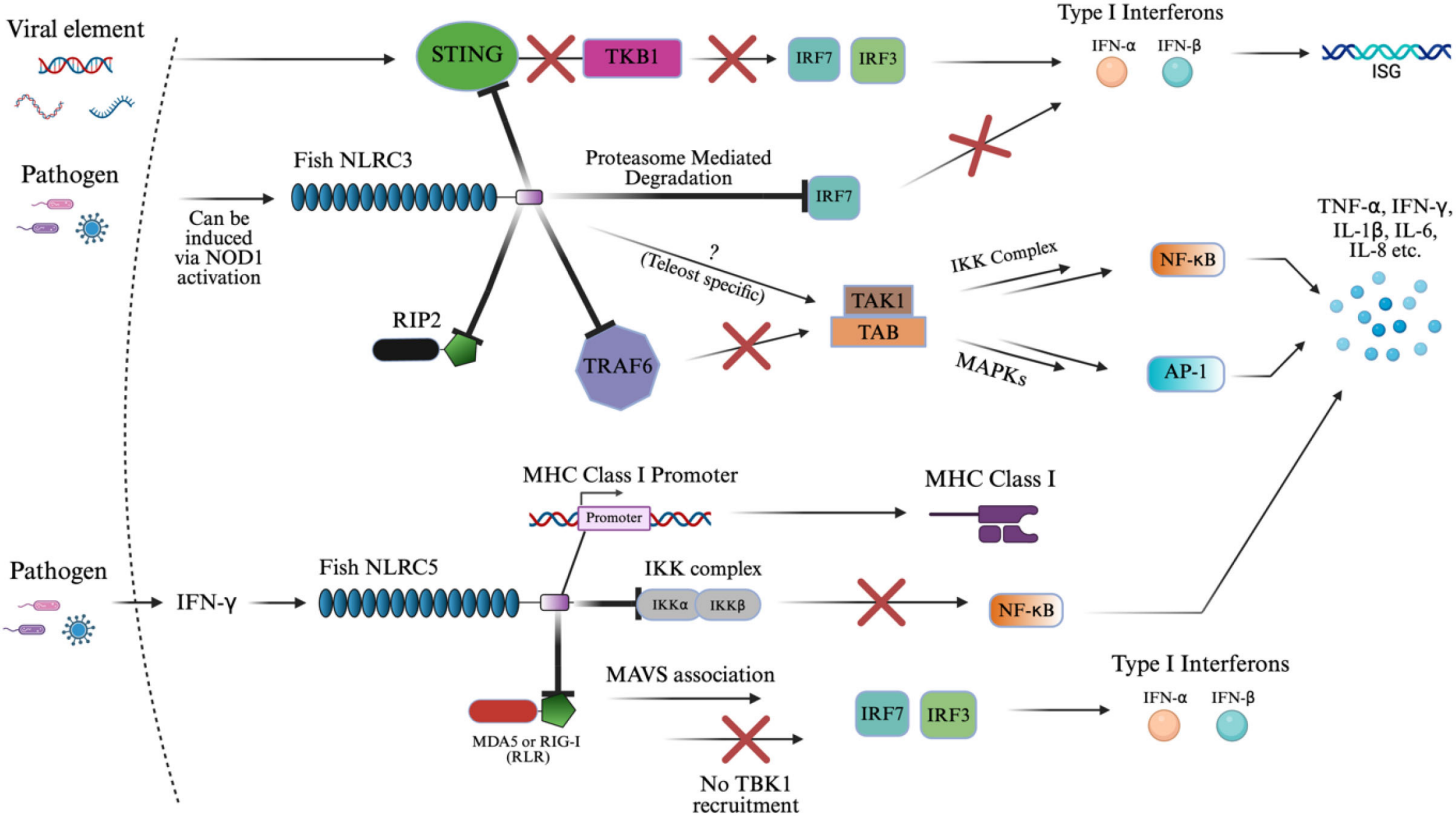
NLRC3 and NLRC5 signaling activation of innate immunity. The schematic representation of NLRC3 and NLRC5 signaling pathways in immune regulation. This diagram reflects the known inhibitory roles of NLRC3 in IFN-I, NF-κB, and AP1 signaling via suppression of STING-TKB1, TRAF6, RIP2, and IRF-mediated pathways. The “X” symbol in red represents some form of inhibition or suppression of the subsequent biomolecule. The “?” symbol represents an unknown pathway where teleost-specific interactions may differ by contributing to cytokine upregulation in fish species. Similarly, NLRC5 presents a similar result by downregulating cytokine production downstream of NF-κB and IFN-I response through inhibitory binding to the IKK complex and RLRs (MDA5 or RIG-I), while also directly promoting MHC class I expression via binding to the MHC class I promoter. The image was created using BioRender.com.

**Figure 5** summarizes the inhibitory signaling roles of NLRC3 and NLRC5, which are described in detail below. NLRC3 is broadly inhibitory across multiple inflammatory axes. In a key mechanism in large yellow croaker, NLRC3 directly binds to the adaptor protein, STING, inhibiting its binding to TBK1 in the presence of viral RNA or DNA (27, 168, 169). This attenuates their ability to activate IRF3/IRF7 promoters, which in turn limits IFN-I production (27, 168, 169). This mechanism parallels findings in mammalian models and underscores a conserved inhibitory strategy within vertebrates. By blocking this pathway, NLRC3 reduces downstream expression of ISGs (169). In addition, NLRC3 promotes proteasome-mediated degradation of IRF7, further dampening IFN-I (IFN-α/β) response (170).

NLRC3 also acts on NF-κB and AP-1 signaling by binding to or interacting with critical upstream components such as RIP2 and TRAF6 (87, 137, 168, 169). In teleosts, overexpression of NLRC3 binding to TRAF6 selectively suppressed K63-linked ubiquitination, required for signaling, and enhanced K48-linked ubiquitination of TRAF6, promoting its proteasomal degradation. As a result, TRAF6 abundance in the cytoplasm was reduced, preventing the assembly of the TAK1–TAB complex, which is necessary for activating both the IKK complex (leading to NF-κB nuclear translocation) and MAPKs (activating AP-1) as outlined in **section 3.1** (87, 137, 168, 169). In addition to TRAF6, piscine NLRC3 also interacts directly with RIP2 via its NACHT domains (171). This binding interferes with the formation of the NOD1–RIP2 complex, thereby suppressing NOD1-mediated activation of NF-κB and MAPKs (171). Overexpression of zebrafish NLRC3 not only blocked this signaling axis but also downregulated the transcription of genes encoding key cytokines and chemokines during bacterial infection (137, 171). In contrast, in goldfish, NLRC3 interacts with RIP2 without binding to suppress NF-κB, AP-1, and IFN-I activity, suggesting species-specific variation in regulatory mechanisms (137, 172).

Interestingly, NOD1 and RIP2 can act as transcriptional regulators of NLRC3, downregulating these proteins in the presence of NLRC3 in zebrafish (137, 171, 173, 174). Also, the pathway may be modulated differently in teleost fish, where NLRC3 was associated with the upregulation of pro-inflammatory cytokines, indicating possible teleost and pathogen-specific pathways, which are not completely understood (137). This could result in a similar potential to NOD1/2 as a stimulator of adaptive immunity.

NLRC5 similarly inhibits the NF-κB and type I interferon pathways, but through distinct molecular interactions. Instead of acting upstream, NLRC5 binds directly to the IKK complex, blocking its ability to phosphorylate IκB and thereby preventing the release and nuclear translocation of NF-κB (72, 175, 176). This suppression of NF-κB results in the downregulation of several cytokines in teleost mucosal and systemic tissues. Additionally, NLRC5 inhibits RLRs such as MDA5 and RIG-I, resulting in MAVS association that interferes with TBK1’s recruitment of IRF3 and IRF7, suppressing IFN-I production (72, 154, 175). In zebrafish, this function appears to be IFN-independent, as overexpression of NLRC5 reduced viral replication of SVCV (spring viremia of carp virus) without activating type I IFN promoters, distinguishing it from classical antiviral PRRs (175).

A unique feature of NLRC5 is its dual role in immune regulation. In addition to suppressing inflammation, it has a transcriptional regulatory capacity (27, 75, 175, 177). In mammals, NLRC5 translocates to the nucleus via a bipartite nuclear localization signal (NLS) and acts as a master transactivator of MHC class I genes through interaction with the SXY module of the MHC I enhanceosome (175, 178–182). In contrast, zebrafish NLRC5 contains a monopartite NLS and shows a nuclear-cytoplasmic distribution. It was even found that microinjected larvae of zebrafish selectively activate MHC class II genes, substituting CIITA’s function, resulting in a unique discovery (175, 178, 179). NLRC5 in teleosts can translocate into the nucleus and bind directly to the MHC class I promoter, facilitating its expression in response to IFN-γ (27, 75, 175, 177). This enhances antigen presentation and supports adaptive immune responses, distinguishing NLRC5 from other NLRs, which typically lack transcriptional regulatory functions.

### 3.3 NLRX1 and NLRP1

**Figure 6:**
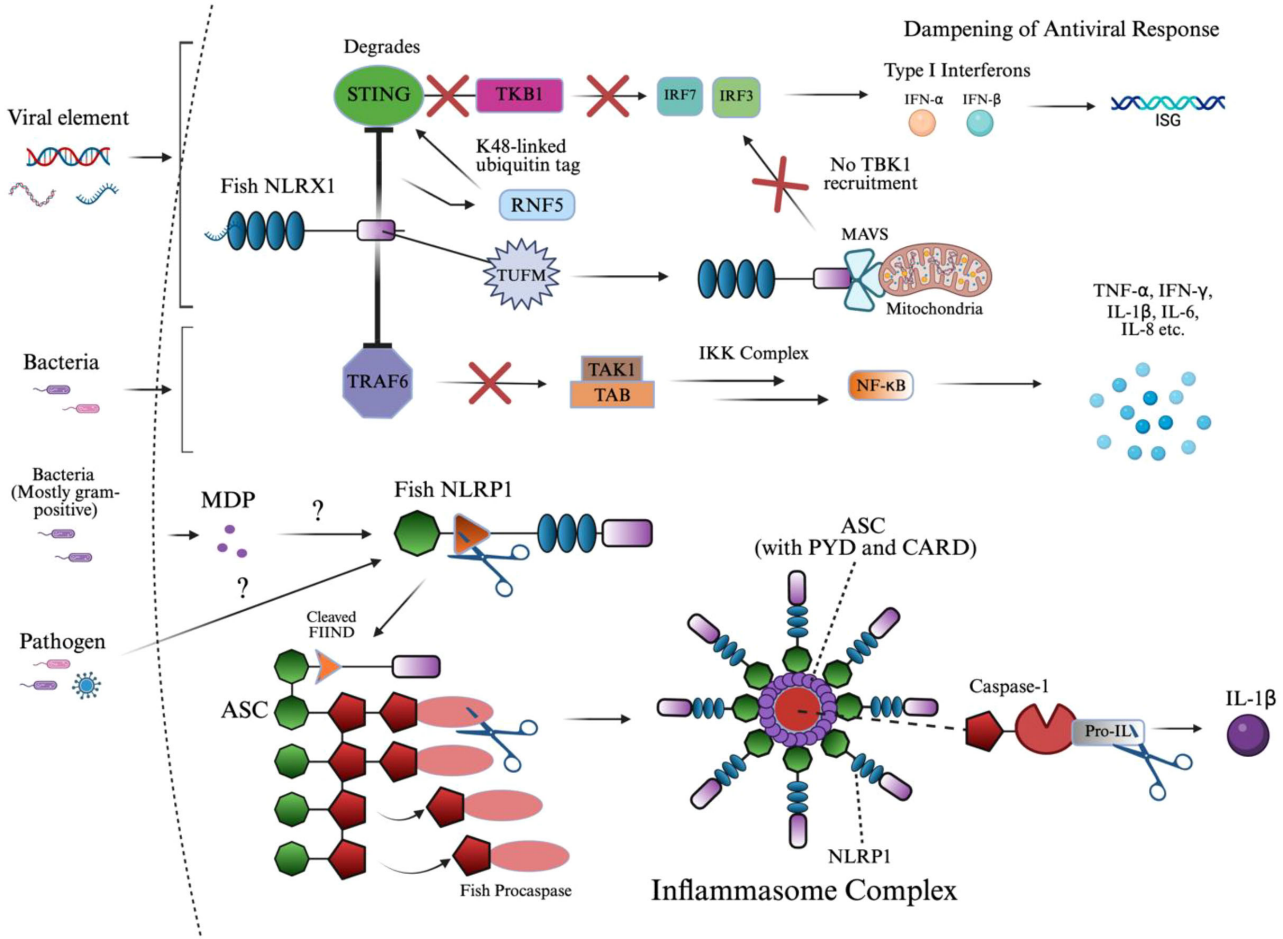
NLRX1 and NLRP1 signaling activation of innate immunity. This diagram illustrates the dual role of fish innate immune sensors NLRX1 and NLRP1 in suppressing antiviral signaling and assembling the inflammasome complex, respectively. Upon viral infection, NLRX1 inhibits interferon responses by targeting STING for RNF5-mediated K48-linked ubiquitination and proteasomal degradation, thereby preventing TBK1 recruitment and downstream IRF activation. Similarly, TUFM enhances NLRX1-mediated inhibition of MAVS signaling by stabilizing the mitochondrial complex, parallelly dampening IRF3/7 phosphorylation and IFN-I production. In the context of bacterial infection, NLRX1 directly interacts with TRAF6 through its NACHT domain, preventing activation of the TAK1–TAB–IKK complex and downstream NF-κB signaling, ultimately reducing the expression of pro-inflammatory cytokines. In parallel, MDP or unknown pathogen-associated ligands are proposed to activate fish NLRP1, which undergoes FIIND domain cleavage and associates with ASC. This leads to the formation of a filamentous inflammasome complex, which recruits and activates caspase homologs through PYD–PYD interactions, enabling the cleavage of pro-IL-1β into its mature, secreted form IL-1β. The structure emphasizes domain architecture and signaling convergence between viral and bacterial response mechanisms in teleosts. The image was created using BioRender.com.

**Figure 6** outlines the distinct signaling mechanisms of NLRX1 and NLRP1, which are detailed below. NLRX1 was shown to act similarly to NLRC3 via its slightly varied inhibition of STING to TBK1 binding, and inhibition of NF-κB via TRAF6 (11, 169, 183). Upon viral infection, the zebrafish NLRX1 isoform was shown to downregulate IFN response by targeting STING for proteasome-dependent degradation (11). Mechanistically, NLRX1 binds to the N-terminal domain of STING and recruits the E3 ubiquitin ligase RNF5, which catalyzes K48-linked polyubiquitination of STING and marks the protein for proteasome degradation (11). As a result, STING protein levels are reduced before it can interact with and activate TBK1, blocking the phosphorylation of IRFs and the production of antiviral IFNs (11).

Much like NLRC5, antiviral signaling also begins with RIG-I or MDA5 recognizing viral RNA and activating MAVS (184, 185). Under normal conditions, MAVS oligomerizes and recruits downstream kinases TBK1 and IKKϵ, which phosphorylate IRF3/7, leading to their nuclear translocation and the induction of IFNs and ISGs (185). However, in black carp, this signaling is negatively regulated by the mitochondrial protein NLRX1, as the protein prevents MAVS oligomerization upon binding at the mitochondrial membrane and NACHT domain, blocking recruitment of TBK1 (185, 186). TUFM, another mitochondrial-associated protein, does not bind MAVS directly but instead associates with NLRX1 to enhance its inhibitory effect, collectively dampening the antiviral IFN response and promoting viral persistence (185).

During *Edwardsiella piscicida* infection in zebrafish, NLRX1 overexpression led to increased bacterial proliferation and decreased host survival (183). Mechanistically, NLRX1 interacts directly with TRAF6 at its NACHT domain, inhibiting TRAF6’s ability to activate downstream NF-κB signaling (183). This resulted in the negative regulation of pro-inflammatory cytokines, IL-6, IL-8, TNF-α, and IL-1β, and specific antimicrobial peptides in a very similar manner to NLRC3 (169, 183).

The ligand responsible for NLRP1 activation has been identified as MDP in fish, along with an imbalance of the redox state in humans (28, 101). The NLR uses its FIIND domain, which is involved in autolytic cleavage, to induce a conformational change of the CARD domain to interact with the CARD of ASC, as referenced in section 2.6 (28, 187–191). This FIIND domain in NLRP1 consists of the ZU5 and UPA subdomains in humans, where ZU5 is cleaved, initiating the signaling cascade; however, in fish, the structure of the domain has not been identified (192). Moreover, these homotypic interactions of ASC in humans are replaced by PYD-PYD interactions, which fish lack (187–191). Due to the homology between these domains in signaling ASC, it was found that the PYD domain was dispensable in humans (132, 187). Both mechanisms of NLRP1-ASC binding result in nucleation of a filamentous platform for procaspase binding.

As mentioned, procaspase-A and procaspase-B were identified in zebrafish and are likely homologs of procaspase-1, which is the canonical NLRP1 inflammasome protein across most studied species (28, 193–196). Other, nonconventional proteins have been identified in humans, caspase-4 and caspase-5, and in mice, caspase-11. The specific type of procaspase or caspase will not be mentioned due to the complexity of species-specific variation involved in teleosts, and should be implied as caspase-1 or its homologs. In zebrafish and common carp, the caspase precursor, procaspase, contains a PYD domain instead of the canonical CARD domain seen in mammals (28, 101, 187, 193). This domain substitution is critical, as it alters the recruitment mechanism: instead of CARD–CARD binding, teleost ASC filaments interact with procaspase via PYD–PYD binding, driving oligomerization to form the inflammasome (28, 101, 187, 193). This distinction in procaspase structure indicates that some or all teleosts may have undergone lineage-specific modifications to accommodate different innate immune contexts.

Structurally, the activated inflammasome in studied teleosts forms a three-dimensional ring-like complex following the cleavage of procaspase (28, 101, 193). This domain architecture is composed of multiple NLRP1 molecules branched to ASC filaments that form the core (28, 101, 193). Procaspase molecules are anchored to these ASC filaments, which cannot be visualized in a top-down view, allowing for efficient clustering and activation (28, 101, 193). The integrity of this oligomerized structure is crucial for the spatial coordination of signal transduction, ensuring that cytokine signaling is regulated and localized.

Procaspase is then cleaved within the inflammasome at conserved aspartate residues between its large (subunit varies) and small (often p10) catalytic subunits, forming the active caspase heterodimer, identified as p35 in zebrafish (28, 197). Once activated, caspase cleaves pro-inflammatory cytokine, pro-IL-1β, in zebrafish into its mature form, IL-1β (28). In mice, IL-1β and IL-18 are induced following NLRP1 inflammasome formation (198).

Although NLRP3 is not expanded on in this study, the protein has been well studied in several teleosts as an inflammasome that results in inflammatory caspase activation and interleukin-1β maturation, much like NLRP1, and has also been explored in zebrafish, common carp, Atlantic salmon, goldfish, and other teleosts (28, 172, 193, 199–202). NLRP3 can coordinate caspase activation in a two-step manner and release IL-18, unlike NLRP1, with both ASC-dependent and ASC-independent (via direct caspase-B triggering) routes (28, 199). The result is linked to gasdermin E-mediated pyroptosis, indicating broader or more flexible downstream effector engagement (199). Despite its importance, NLRP3 is not found in all teleost lineages, and most studies focus on cyprinid species. Still, its similarities to mammalian inflammasome responses make it a valuable model for studying inflammation and immune activation in fish.

## 4 Functional roles of fish NLRs in disease protection

NLR, along with other novel PRRs, can be induced by any pathogen that stimulates the innate immune system, such as bacterial, viral, and parasitic infections, due to the induction of signaling molecules from the presence of specific ligands. These infections have significant implications in aquaculture, where immune dysfunction or overactivation can lead to mortality and economic loss. Additionally, studying fish responses to these pathogens can provide broader insights into vertebrate immune evolution and adaptation.

In this section, bacterial infections will focus on gram-negative and gram-positive bacteria due to their association with iE-DAP and MDP. Regarding viral infections, ssRNA, dsRNA, and dsDNA viruses will also be expanded on. Although ssDNA viruses exist, they will not be discussed in this section due to limited research on their significance in NLR stimulation. For parasitic infections, the discussion will center on protozoan, ectoparasitic, and endoparasitic challenges that have been documented to modulate NLR responses in fish species. The phenotypic traits and genetic elements of these pathogens will be emphasized as they are key to determining the class of NLR stimulated via differences in ligands presented. Bacterial features like lipid A structure and motility, as well as TLR activation, lie beyond the scope of this analysis but warrant future investigation. Similarly, the location of tissue upregulation will not be a point of emphasis in terms of organization due to the variety of teleost species and NLRs covered. It should be noted that NOD1/2 are conserved across tissues, and are especially prominent in the mucosal barriers of gills, skin, and intestinal mucosa, where they provide cellular and humoral protection through the release of cytokines and recruitment of T cells (203). **Table 1** summarizes the diversity of NLR ligands and pathogen interactions across bacterial, viral, and parasitic infections in teleost fish.

**Table 1:** Pathogen and ligand diversity driving NLR signaling pathways in teleost fish.

NLR	Known ligands	Bacterial fish pathogens activation	Viral fish pathogens activation	Protozoan fish pathogens activation
NOD1	iE-DAP (95), LPS (204), viral dsRNA (143)	*Edwardsiella piscicida* (149, 205), *Flavobacterium columnare* (149), *Aeromonas hydrophila* (94, 96, 206)*, Streptococcus agalactiae* (97), *Edwardsiella ictalurid* (93), *Shigella flexneri* (55), *Edwardsiella tarda* (94, 206), *Streptococcus uberis* (96), *Vibrio anguillarum* (99), *Mycobacterium marinum* (98), *Aeromonas salmonicida* (98), *Streptococcus iniae* (206)	Nervous necrosis virus (NNV) (207), Tilapia lake virus (TiLV) (97), Grass carp reovirus (GCRV) (208–211), catfish hemorrhage reovirus (CCRV) (206), Poly (I:C) (143)	*Cryptocaryon irritans* (212)
NOD2	MDP (Muramyl dipeptide) (213), viral ssRNA (153)	*Edwardsiella piscicida* (205), *Aeromonas hydrophila* (96, 206), *Edwardsiella tarda* (55, 206)*, Vibrio anguillarum* (99), *Streptococcus agalactiae* (97), *Edwardsiella tarda* (55, 206), *Streptococcus uberis* (96), *Vibrio anguillarum* (99), *Mycobacterium marinum* (98), *Aeromonas salmonicida* (98), *Streptococcus iniae* (206)	TiLV (97), GCRV (208–211), CCRV (206)	NA
NLRC3	Viral RNA (214), viral DNA (84), LPS (214)	*Aeromonas hydrophila* (206), *Edwardsiella tarda* (100, 206, 215), *Streptococcus agalactiae* (97)*, Streptococcus iniae* (100, 206, 215)	Hirame novirhabdovirus (HIRRV) (216), hematopoietic necrosis virus (IHNV) (217), spring viremia of carp virus (SVCV) (137, 170), Salmon Anemia Virus (ISAV) (218), Piscine orthoreovirus-1 (PRV-1) (219), CCRV (219), Poly (I:C) (214)	*Ichthyophthirius multifiliis* (220)
NLRC5	Viral RNA (221), LPS (175)	*Aeromonas hydrophila* (206), *Edwardsiella tarda* (206), *Streptococcus iniae* (101, 206)	IHNV (217), ISAV (218), CCRV (206), Poly (I:C) (221)	*Cryptocaryon irritans* (212)
NLRP1	Viral RNA and DNA (28), MDP (28, 101)	*Aeromonas hydrophila* (101)*, Edwardsiella tarda* (28)	ISAV (218)	NA
NLRX1	Viral RNA and DNA (11, 185), LPS (186)	*Aeromonas hydrophila* (98, 206), *Aeromonas salmonicida* (98), *Mycobacterium marinum* (98), *Streptococcus iniae* (206), *Edwardsiella tarda* (206)	SVCV (11), CCRV (206)	*Paramoeba perurans* (222)
NLRs (not identified)	Unknown	NA	TiLV (223, 224), Piscine myocarditis virus (PMCV) (225, 226), Megalocytivirus (MCV) (227–230), CCRV (206)	*Cryptocaryon irritans* (231), *Ichthyophthirius multifiliis* (220), *Amyloodinium ocellatum* (232, 233)

### 4.1 Bacterial infections

The functional roles of NLRs upon bacterial infection in fish can be pro-inflammatory or regulatory, depending on the receptor type. Regarding pro-inflammatory defense, NOD1 was shown to elicit the expression of IL-1β, IL-8, and other antimicrobial effectors in zebrafish (*Danio rerio*) upon administration of *Edwardsiella piscicida* (edwardsiellosis), promoting NF-κB and MAPK pathway activation through RIP2 interaction (149). This same study showed that *Flavobacterium columnare* had a similar effect (149). Similarly, an *E. piscicida* vaccine was constructed that substituted the chromosomal *murA* promoter with the arabinose-dependent *araC* P_araBAD_ cassette (205). Removal of this arabinose upon administration resulted in cell wall lysis and spread of the strain, upregulating TNF-α, IL-1β, IL-8, IL-6, and IFN-γ by stimulating both NOD1 and NOD2 in channel catfish (*Ictalurus punctatus*) (205). In this same fish species, NOD1 expression in the intestine increased ~6-fold at 3 days post-infection with *Edwardsiella ictaluri* (93). Similarly, three bacterial infections of *Aeromonas hydrophila, Shigella flexneri*, and *Edwardsiella tarda* upregulated NOD1 and RIP2, forming a complex for further signaling in rohu (94). It should be noted that *Shigella* spp. do not present virulence activity below 35°C, which limits its pathogenic potential in ectothermic hosts such as most fish, but it was worth mentioning (234). The mentioned bacterial species are all gram-negative bacterial infections, resulting in the release of the iE-DAP bacterial ligand in NOD1 specifically, as outlined in **Figure 4** (56, 93, 96, 149, 205). Gram-positive bacteria can also upregulate NOD1 upon iE-DAP stimulation with *S. agalactiae* infection in Nile tilapia (*Oreochromis niloticus*) as well as *Streptococcus uberis* in mrigal (*Cirrhinus mrigala*), which increased NF-κB and subsequent pro-inflammatory cytokine production (96, 97).

Gram-negative bacteria still contain a peptidoglycan, releasing MDP in some cases for NOD2 ligand binding (54). As a result, in rohu (*Labeo rohita*), *E. tarda* infections upregulated NOD2 ~5-6-fold, inducing IFN-γ production up to ~10-fold (55). This same study found rohu infected with *A. hydrophila* upregulated NOD2 from ~2 to ~5-fold, while infected mrigal (*Cirrhinus mrigala*) upregulated both NOD1 and NOD2, conferring the production of IL-8, IL-1β, and IFN-γ, primarily in the liver, kidney, and spleen (96). Similarly, miiuy croaker (*Miichthys miiuy*) infected with *Vibrio anguillarum* resulted in varying expression levels of NOD1 and NOD2 in these same organs (99). Concerning the same ligand pathway in gram-positive bacteria, *S. uberis* infection in migral resulted in an even greater NOD2 upregulation than the previously mentioned gram-negative bacteria due to the increased presence of MDP (96).

Bacterial infections also activate other NLRs, either individually or in combination, which vary depending on fish and bacterial species. *E. tarda* and *Streptococcus iniae*, which are Gram-negative and Gram-positive bacteria, respectively, resulted in the expression of NLRC3 in Japanese flounder (*Paralichthys olivaceus*), subsequently increasing IL-1β mRNA expression (100, 215). Likewise, a similar study induced *E. tarda, A. hydrophila, and S. iniae* infections in channel catfish, resulting in the upregulation of NOD1, NOD2, NLRC3, NLRC5, and NLRX1 in the intestine, liver, and head kidney, along with varied instances of NLR downregulation in the spleen (206). NOD1, NOD2, and NLRX1 were induced in goldfish (*Carassius auratus* L.) upon heat-killed *Aeromonas salmonicida* and the acid-fast bacterium *Mycobacterium marinum* challenges, indicating functional conservation of NLRs in teleost fish (98). In a previously mentioned study, *S. agalactiae* underwent MDP-stimulation, overexpressing NOD2 and NLRC3, which peaked in the spleen, kidney, gill, and blood of Nile tilapia, enhancing NF-κB signaling (97). *E. tarda* and *A. hydrophila* infections were also shown to upregulate NLRP1 expression in common carp (*Cyprinus carpio*) (101). *E. tarda* had the same effect in zebrafish, activating caspase-A/B, functional homologs of caspase-1 in mammals, and IL-1β, indicating inflammasome activation (28). The further signaling pathway of pro-inflammatory or regulatory response was not always performed; however, it should be noted that NLRC3, NLRC5, and NLRX1 have been shown to result in inhibitory effects on innate immune signaling pathways in fish, as mentioned (11, 137, 235). Direct LPS (Lipopolysaccharide), peptidoglycan (PGN), MDP, and iE-DAP have been administered *in vitro* and *in vivo*, resulting in similar NLR production, further backing these mechanisms (96, 204). Several other studies using other bacterial and fish species combinations have been performed, with the general principles of NLR signaling applied upon infection.

### 4.2 Viral infections

Devastation to aquaculture has been largely driven by viral infections. As a result, extensive research has been done in teleosts to find downstream immunogenic signaling proteins and cytokines. Their variability in ssRNA, dsRNA, and dsDNA forms makes it especially challenging for the novel prediction of immune response and elicits a wide range of NLRs, though distinctions between positive-sense and negative-sense RNA viruses are beyond the scope of this review.

Several ssRNA viruses have induced NLR response in various teleosts. One study in orange-spotted grouper (*Epinephelus coioides*) found that NOD1 was slightly upregulated following infection with the nervous necrosis virus (NNV), but acts as a negative modulator of IFN signaling at the RNA sensor level by suppressing RIG-I- and MDA5-mediated IFN promoter activation (207). In Nile tilapia, Tilapia lake virus (TiLV) infection activated NOD1 and NOD2, such as TRAF-mediated NF-κB activation, which induces pro-inflammatory cytokines, particularly IL-1β, which, together with viral viroporins, promotes NLRP3 inflammasome formation (97, 223, 224, 236–238). Also, the Hirame novirhabdovirus (HIRRV) in Japanese flounder upregulated NLRC3 and several interferon-stimulated genes, including IRF3, IRF7, IKKβ, and TBK1 (216). Signaling became broader when rainbow trout infected with hematopoietic necrosis virus (IHNV) significantly upregulated NOD1, NLRC3, and NLRC5 expression in their skin, alongside pro-inflammatory cytokines such as IL-1β, IL-6, IL-8, TNF-α, and IFN-I (217). NLR studies of spring viremia of carp virus (SVCV) were tested in several teleosts where species-specific immune modulation occurred (137, 170). In zebrafish, NLRC3-like proteins act as either positive or negative regulators during pathogen infection, while in grass carp, NLRC3 acts as a negative regulator by degrading IRF7 and suppressing the RLR-mediated interferon response, ultimately enhancing viral replication (137, 170). Along with this, SVCV upregulates NLRX1 in zebrafish and suppresses the IFN response by degrading and disrupting STING–TBK1 signaling (11). In Atlantic salmon infected with Infectious Salmon Anemia Virus (ISAV), NLRC3, NLRC5, and NLRP1 were upregulated in gills and head kidney alongside interferon-stimulated genes and pro-inflammatory cytokines (218). The specific regulatory effects on innate immunity of these three NLRs were not further explored in this study (218).

Similarly, the effects of dsRNA viruses have also been observed in grass carp infected with grass carp reovirus (GCRV), where NOD1 and NOD2 expression was significantly upregulated in the spleen and trunk kidney (208–211). In Atlantic salmon red blood cells exposed to Piscine orthoreovirus-1 (PRV-1), NLRC3-like receptors were primarily expressed (219). Likewise, a broader study focused on the outcomes of Atlantic salmon suffering from cardiomyopathy syndrome (CMS) following intraparietal injection of piscine myocarditis virus (PMCV) in the heart, displayed the general upregulation of NLRs (225, 226). The last notable finding was the production of NOD1, NOD2, NLRC3, NLRC5, and NLRX1 from a previously mentioned bacterial study that also introduced a channel catfish hemorrhage reovirus (CCRV) challenge (206). None of these dsRNA virus studies explored any form of downstream immune regulation mediated by these NLRs. Also, upon polyinosinic:polycytidylic acid (poly (I:C)) challenge, NLRC5 was upregulated, and failed to activate IFN-Is (239).

The last classification of piscine viruses is composed of dsDNA, such as megalocytivirus (MCV) administered in spotted knifejaw (*Oplegnathus punctatus*), which presented general NLR expression, while channel catfish virus (CCV) elicited NLR, RIG-I, and MDA5, constitutively expressed across different tissues (227–230). There are several other ssRNA, dsRNA, and dsDNA viruses studied or are yet to be studied that may play a pivotal role in identifying a canonical ligand or signaling pathways to predict NLR expression.

### 4.3 Parasitic infections

In contrast with bacterial and viral pathogens, parasites typically develop long-term relationships with their hosts, testing the immune system in novel ways. Interactions with NLRs in response to infection vary with patterns of fish and parasite species but may act as a key driver for resistance in teleosts. Research on NLR response following parasitic infection in fish has been sparsely addressed, primarily focusing on protozoan, ectoparasite, and endoparasitic challenges *in vivo*.

*Cryptocaryon irritans is* an obligate ciliate parasite that embeds itself in the epithelial tissue of marine fish species, leading to white spot lesions and secondary infections (240). Exposure to *C. irritans* in golden pompano *(Trachinotus ovatus)* led to elevated expression of APAF1 and NOD1 in nearby skin regions (NRS), while NLRC5 was downregulated in that region, suggesting increased apoptotic and inflammatory activity (212). Another study in Japanese pufferfish (*Takifugu rubripes*) infected with the same parasite also revealed NLR induction (231). On the other hand, the ciliate protozoan parasite, *Paramoeba perurans*, makes surprising attempts to possibly even evade innate immune responses in Atlantic salmon by downregulating NLRX1 along with IL-1β, TNF-α, IFNα3, and IRFs (222).

In grass carp infected with *Ichthyophthirius multifiliis*, a bacterium causing white spot disease in freshwater fish, significantly upregulated NLRC3 and NLRP3 in resistant individuals, contributing to pathogen recognition and resistance by suppressing NF-κB signaling to prevent excessive inflammation (220). Another study found that following an *Amyloodinium ocellatum* infection, the inflammasome, NLRP12, and other NLR components, including CARD9 and Proline-Serine-Threonine Phosphatase Interacting Protein 1 (PSTPIP1), were significantly upregulated in the skin of golden pompano (232, 233). While studies on parasite-induced NLR activation in fish remain limited, mammalian research has demonstrated a wide range of NLRs, such as NOD1, NOD2, NLRP1, NLRP3, and NLRP12, which are activated in response to protozoan infections of *Entamoeba histolytica, Leishmania* spp.*, Plasmodium* spp.*, Toxoplasma gondii*, and *Trypanosoma cruzi* leading to inflammasome assembly, cytokine production, and modulation of Th1/Th2 responses in mouse, human epithelial, monocytic cells, and in rats (201, 241–257).

## 5 Emerging role of NOD-like receptors in vaccine-induced immunity in aquaculture

As aquaculture continues to expand globally, effective vaccination strategies remain essential for disease control and fish health management. While many current vaccines rely on surface PRRs such as Toll-like receptors (TLRs) for immune activation, recent research suggests that intracellular sensors like NLRs may also play a role in modulating vaccine-induced innate and adaptive immunity, possibly through pathways outlined in section **3**. Disease outbreaks pose a major threat to the rapidly growing aquaculture industry, and effective vaccination is crucial for sustainable production.

Vaccination exposes fish to antigenic components of a pathogen to trigger a protective immune response to prevent disease upon subsequent exposure. They are often coupled with an adjuvant to boost innate immune activation and antigen presentation, and with a delivery vector to enhance cellular uptake, stability, and targeting. Adjuvants often contain known bacterial ligands which have the potential to engage with fish NLRs, especially NOD1 and NOD2 to encourage the previously mentioned pathways of immune response.

### 5.1 Adjuvants and vaccines as NLR agonists

NLRs, like other PRRs, are not used as adjuvants themselves due to several challenges. This includes their intracellular, cytosolic presence, risk of hyperinflammation, as there may be overexpression of cytokine response, and impracticality, as activation through known NLR activating ligands is more ideal. Mammalian studies have shown that NLR upregulation follows the administration of adjuvants and vaccines serving as analogs of canonical ligands, such as iE-DAP and MDP, eliciting NOD1 and NOD2 activation, respectively. This can trigger signaling pathways inside cells that influence cytokine production and antigen presentation. These systems are better characterized in mammals due to more extensive immune cell profiling and transgenic animal availability. Although there is currently no direct evidence that NLRs serve as immune targets in developing aquaculture adjuvants in fish, new studies hint at their possible role in shaping host immune responses. Therefore, we suggest NLRs as promising, though unverified, candidates for immunomodulation in aquaculture.

In mammals, MDP-containing adjuvants such as Complete Freund’s Adjuvant (CFA), Murabutide, and Muramyl-Tripeptide Phosphatidylethanolamine (MTP-PE) have been shown to elicit NOD2, and even in some cases NOD1 (258–264). Notably, MDP was identified as a key component of CFA’s adjuvancy, where NOD2 is required for effective CD4+ T cell priming, resulting in immunoglobulin G (IgG1 and IgG2c) production (258, 260, 265). The MDP synthetic derivative, Murabutide, was shown to activate HIV-infected antigen-presenting cells and induce cytokines that suppress viral replication, further demonstrating NOD2’s immunomodulatory potential (258, 261, 262). Similarly, MTP-PE reduced pyrogenicity and significantly enhanced cellular immunity (258, 263, 266). It should be noted that *N*-acetyl MDP is produced by most bacteria and activates NOD2; however, *N*-glycolyl MDP is produced by the NamH enzyme in *Mycobacterium* and has been found to evade NOD2 signaling in mammals, which most likely also applies to fish (260). DAP containing adjuvants also exist, signaling NOD1, such as CFA and the synthetic peptides, FK-156 and FK-565. In CFA, DAP-type PGN fragments exist, promoting Th1 polarization and class switching to IgG2b, IgG2c, and IgG3 antibody isotypes, while FK-156/565 induced a Th2-biased immune response in human dendritic cells (264, 265, 267). To effectively introduce NOD ligands, encapsulation using poly(lactic acid) nanoparticles has been used and added with the antigenic components of a vaccine to produce NOD1 and NOD2 activation (258, 268). In fish, these adjuvants have also been explored extensively and have been clearly shown to elicit cytokine and immune responses. While direct demonstration of NOD1/2 activation remains limited in fish, the conservation of NLR signaling in fish studies from other methods of iE-DAP and MDP-based challenges suggests similar effects by these adjuvants (269, 270). In mammals, several adjuvants were also shown to act as NLRP3 agonists, resulting in a similar inflammasome cascade as NLRP1, producing IL-1β and IL-18 (258, 271).

Although the role of NLRs in fish immunity is still emerging, their potential as adjuvant targets spans several vaccine platforms. Live attenuated and DNA vaccines may naturally engage NLRs through intracellular PAMPs, while inactivated and subunit vaccines could benefit from co-administered NLR agonists, like the ones mentioned, potentially enhancing cytokine production, APC maturation, and T or B cell responses. However, studies directly assessing NLR activation by these different vaccine platforms are limited. Recent literature has demonstrated that recombinant attenuated *Edwardsiella piscicida* vaccines (RAEVs) activate NOD1- and NOD2-mediated signaling pathways in teleost fish, resulting in the upregulation of pro-inflammatory cytokines and robust innate immune responses that confer protection against wild-type *E. piscicida* challenge (205). These findings align with recent reviews highlighting the importance of NOD-like receptor pathways in modulating mucosal and systemic immune responses to *Aeromonas hydrophila* vaccines in fish, particularly under immersion and oral delivery conditions (272). Some notable previously mentioned studies include a heat-killed bacterial challenge in goldfish and a live-attenuated, recombinant, *Edwardsiella piscicida* vaccine in channel catfish (98, 205). The first study resulted in the upregulation of NOD1, NOD2, and NLRX1, as mentioned, with no further downstream analysis, while the second produced IL-8, IL-1β, TNF-α, IL-6, and IFN-γ following the NF-κB pathway from NOD1 and NOD2 activation (98, 205). Along with these, a formalin-inactivated *Vibrio anguillarum* vaccine tested in Japanese flounder resulted in upregulation of NOD1, NOD2, NLRC3, and NLRC5, along with key proteins involved in the cascade to produce cytokines (13). In this same species, strictly NLRC5 was tested upon formalin-killed *E. tarda* and *S. iniae* infections, and was significantly upregulated from only the *E. tarda* vaccine (239).

## 6 Challenges and future directions in aquaculture

As the aquaculture industry continues to face evolving pathogen challenges and production demands, recent innovations in fish vaccination have focused on improving antigen design, adjuvant efficacy, and mucosal delivery strategies (273). These advances underscore the importance of integrating host-pathogen interaction studies with practical immunization platforms to achieve sustainable disease management (273). Despite the advances in the knowledge about fish NLRs, several gaps continue to restrict their use in aquaculture. One such challenge is the partial functional characterization of many teleost NLRs. The unique structural domains and context-dependent processes of mammalian homologs in fish make it difficult to make direct comparisons. In this regard, teleost-specific subfamilies such as within NLRC have resulted in inconsistent nomenclature of the protein. Also, evolutionary divergence regarding gene duplications and structural diversity results in species-specific mechanisms for signaling. Notably, adaptations such as the teleost-specific modification of NLRC3, acting as a positive regulator of downstream pro-inflammatory responses, indicate functional divergence from the mammalian counterpart. This highlights the need for both comparative genomics and functional verification in commercially relevant aquaculture models.

Another key obstacle is ligand specificity. While some ligands’ functional roles have been displayed, such as iE-DAP and MDP, many other ligands remain unidentified or untested in fish species. Mapping these ligand-receptor relationships is critical for understanding how different antigenic elements trigger innate responses through NLRs. These efforts would also inform vaccine development, as interspecies variation in NLR expression and signaling presents challenges for adjuvant design. Also, certain NLR agonists may be ineffective or incompatible with specific vaccine formulations, stressing the importance of determining optimal dosages and delivery systems to minimize inflammation or toxicity.

Approaches such as nanoparticle-mediated delivery could also idealize target effects. Along with this, the development of multi-component adjuvant systems combining NLR agonists with ligands for other PRRs may yield synergistic effects, amplifying protective immunity. However, these combinations must be empirically tested in fish models, detailing the presence of cross-talk and downstream signaling cascades in the presence of TLRs, RLRs, etc., to confirm compatibility and efficacy. One of the major challenges in advancing fish vaccine development is the limited understanding of mucosal immune mechanisms and the intracellular PRRs that regulate them. Despite promising data on the involvement of NLRs in pathogen sensing, few studies have systematically explored their role in mucosal immunization or adjuvant response (272).

The central focus of subsequent studies should be the discovery of novel NLR agonists that are safe, efficient, and applicable for aquaculture. Currently, fish studies use several known mammalian NLR agonists as adjuvants; however, these studies lack findings of NLR induction for vaccine use other than the use of the ligand itself. Integrating high-throughput transcriptomic and proteomic approaches may aid in uncovering the implications of these vaccine components, uncharacterized NLR pathways, and their regulators in fish.

## 7 Conclusion

The study of NLRs in fish reveals a remarkably intricate and evolutionarily diverse family of intracellular immune regulators, first identified in mammals. These proteins expanded and diversified structurally in teleosts, likely in response to the unique selective pressures imposed by aquatic pathogens, preceding further divergence in terrestrial vertebrates. Through phylogenetic mapping, structural modeling, and functional characterization, this review illustrates how NLRs in fish retain universal signaling motifs, CARD, NACHT, and LRR domains, which form their nomenclature, while also possessing lineage-specific adaptations that affect ligand recognition, downstream signaling, and immune modulation.

NLRs of fish coordinate a diverse array of activities in response to bacterial, viral, and parasitic infections. NOD1 and NOD2 act as pro-inflammatory sensors by recruiting RIP2 to trigger canonical NF-κB and MAPK cascades. Others, including NLRC3, NLRC5, and NLRX1, serve as immunological brakes, inhibiting IFN-I and inflammatory signaling to avert immune overactivation. Piscine NLRC3 has been shown to also upregulate these cascades in some species, while NLRC5 has a bifunctional role as an MHC-expressional transcriptional regulator and a cytoplasmic inflammatory pathway suppressor. The NLRP1 inflammasome formation displays teleost-specialized adaptations, including unique domain substitutions and mechanisms of caspase activation, further reflecting the structural and functional plasticity of NLR’s gene family in fish.

Even though NLR signaling in parasite and vaccine response has been comparatively less well-explored, new information indicates responsiveness of NLRs to pathogen types and potential to be key determinants of immune resistance. Conservation of central signaling pathways like NF-κB and IRF-mediated cascades, combined with variations in ligand specificity and tissue expression, argues for the application of fish NLRs as targets for immunomodulation to benefit aquaculture. A range of mammalian NLR-agonist adjuvants, with MDP- and iE-DAP–derived compounds leading the list, hold potential in fish. However, the species specificity of NLR expression, localization, and signaling response remains the central challenge.

Future work must prioritize the identification of novel fish-specific NLR ligands, high-throughput screening of NLR-adjuvant interactions, and the development of delivery platforms that target these intracellular pathways without provoking deleterious inflammation. It will be vital to standardize nomenclature, extend functional analysis across commercial teleosts, and combine transcriptomic and proteomic tools such as molecular docking to close the gap between evolutionary insight and vaccine production. Collectively, these endeavors put fish NLRs at the center stage, not only as key innate immunity mediators but potential molecular levers to modulate disease and vaccine efficacy in today’s aquaculture.

## Glossary

NLRA: NLR family acidic transactivation domain containing
NLRB: NLR family BIR domain containing
NLRC: NLR family CARD domain containing
NLRP: NLR family PYD domain containing
NLRX: NLR family with alternative effector domains
NOD1: Nucleotide Binding Oligomerization Domain Containing Protein 1
NOD2: Nucleotide Binding Oligomerization Domain Containing Protein 2
NLRC3: NLR family CARD domain containing protein 3
NLRC4: NLR family CARD domain containing protein 4
NLRC5: NLR family CARD domain containing protein 5
NLRP1: NLR family PYD domain containing protein 1
NLRX1: NLR family member X1
CARD: Caspase Activation and Recruitment Domain
PYD: Pyrin Domain
NB ARC: Nucleotide Binding Apaf 1, R proteins, CED 4 domain
NACHT: Domain named after NAIP, CIITA, HET E, and TP1
LRR: Leucine Rich Repeat
BIR: Baculoviral Inhibitor of Apoptosis Repeat
FIIND: Function to Find Domain
uCARD: Untypical CARD Domain
PRY SPRY: Protein Interaction Domain
AAA+: ATPases Associated with diverse cellular Activities
WHD: Winged Helix Domain
PST: Proline, Serine, and Threonine–rich region
iE DAP: γ D glutamyl meso diaminopimelic Acid
MDP: Muramyl Dipeptide
DAP: Diaminopimelic Acid
LPS: Lipopolysaccharide
PGN: Peptidoglycan
poly I:C: Polyinosinic:polycytidylic acid
Murabutide: Synthetic MDP derivative
MTP PE: Muramyl Tripeptide Phosphatidylethanolamine
FK 156 and FK 565: synthetic diaminopimelic acid–derived peptides
PLA: Polylactic acid nanoparticle delivery
TLR: Toll Like Receptor
RLR: RIG I Like Receptor
CLR: C type Lectin Receptor
PRR: Pattern Recognition Receptor
NF κB: Nuclear Factor kappa light chain enhancer of activated B cells
AP 1: Activator Protein 1
MAPK: Mitogen Activated Protein Kinase
IRF3 and IRF7: Interferon Regulatory Factors 3 and 7
IFN I/IFN α/β: Type I Interferon, alpha/beta
IFN γ: Type II Interferon Gamma
IL 1β: IL 6, IL 8, IL-18, Interleukins 1β, 6, 8
TNF α: Tumor Necrosis Factor Alpha
RIP2: Receptor Interacting Serine/Threonine Protein Kinase 2
TRAF3 and TRAF6: TNF Receptor Associated Factors 3 and 6
MAVS: Mitochondrial Antiviral Signaling Protein
STING: Stimulator of Interferon Genes
TBK1: TANK Binding Kinase 1
TAK1: Transforming Growth Factor Beta Activated Kinase 1
IKKα: IKKβ, IKKγ, IκB Kinase subunits α, β, γ
NEMO: NF κB Essential Modulator
MyD88: Myeloid Differentiation Primary Response 88
MHC: Major Histocompatibility Complex
ROS: Reactive Oxygen Species
NCBI: National Center for Biotechnology Information
BLAST: Basic Local Alignment Search Tool
SMART: Simple Modular Architecture Research Tool
Cryo EM: Cryo Electron Microscopy
RT PCR: Reverse Transcription Polymerase Chain Reaction
ATG: Autophagy Related Gene family
TUFM: Tu Translation Elongation Factor, Mitochondrial
RNF5: Ring Finger Protein 5
CIITA: Class II Transactivator.

## References

[1] Mogensen TH. Pathogen recognition and inflammatory signaling in innate immune defenses. Clin Microbiol Rev. (2009) 22:240–73. doi: 10.1128/CMR.00046-08, PMID: 19366914 PMC2668232

[2] Delbridge LM O’Riordan MXD. Innate recognition of intracellular bacteria. Curr Opin Immunol. (2007) 19:10–6. doi: 10.1016/j.coi.2006.11.005, PMID: 17126540

[3] Sahoo BR. Structure of fish Toll-like receptors (TLR) and NOD-like receptors (NLR). Int J Biol Macromol. (2020) 161:1602–17. doi: 10.1016/j.ijbiomac.2020.07.293, PMID: 32755705 PMC7396143

[4] Li D Wu M. Pattern recognition receptors in health and diseases. Signal Transduction Targeted Ther. (2021) 6:291. doi: 10.1038/s41392-021-00687-0, PMID: 34344870 PMC8333067

[5] Almeida-da-Silva CLC Savio LEB Coutinho-Silva R Ojcius DM. The role of NOD-like receptors in innate immunity. Front Immunol. (2023) 14. doi: 10.3389/fimmu.2023.1122586, PMID: 37006312 PMC10050748

[6] Inohara N Koseki T Lin J del Peso L Lucas PC Chen FF. An induced proximity model for NF-kappa B activation in the Nod1/RICK and RIP signaling pathways. J Biol Chem. (2000) 275:27823–31. doi: 10.1074/jbc.M003415200, PMID: 10880512

[7] Schneider M Zimmermann AG Roberts RA Zhang L Swanson KV Wen H. The innate immune sensor NLRC3 attenuates Toll-like receptor signaling via modification of the signaling adaptor TRAF6 and transcription factor NF-κB. Nat Immunol. (2012) 13:823–31. doi: 10.1038/ni.2378, PMID: 22863753 PMC3721195

[8] Inohara N Koseki T Lin J del Peso L Lucas PC Chen FF. An induced proximity model for NF-κB activation in the nod1/RICK and RIP signaling pathways*. J Biol Chem. (2000) 275:27823–31. doi: 10.1074/jbc.M003415200, PMID: 10880512

[9] Tracey KJ Cerami A. Tumor necrosis factor, other cytokines and disease. Annu Rev Cell Biol. (1993) 9:317–43. doi: 10.1146/annurev.cb.09.110193.001533, PMID: 8280464

[10] Qiao Y Yan W He J Liu X Zhang Q Wang X. Identification, evolution and expression analyses of mapk gene family in Japanese flounder (Paralichthys olivaceus) provide insight into its divergent functions on biotic and abiotic stresses response. Aquat Toxicol. (2021) 241:106005. doi: 10.1016/j.aquatox.2021.106005, PMID: 34731643

[11] Zhao X An L-L Gong X-Y Dan C Qu Z-L Sun H-Y. A zebrafish NLRX1 isoform downregulates fish IFN responses by targeting the adaptor STING. J Virol. (2024) 98:e01801–23. doi: 10.1128/jvi.01801-23, PMID: 38193691 PMC10878056

[12] Ting JP Lovering RC Alnemri ES Bertin J Boss JM Davis BK. The NLR gene family: a standard nomenclature. Immunity. (2008) 28:285–7. doi: 10.1016/j.immuni.2008.02.005, PMID: 18341998 PMC2630772

[13] Liu Y Sheng X Tang X Xing J Chi H Zhan W. Genome-wide identification, phylogenetic relationships and expression patterns of the NOD-like receptor (NLR) gene family in flounder (Paralichthys olivaceus). Fish Shellfish Immunol. (2023) 141:109083. doi: 10.1016/j.fsi.2023.109083, PMID: 37722442

[14] Meunier E Broz P. Evolutionary convergence and divergence in NLR function and structure. Trends Immunol. (2017) 38:744–57. doi: 10.1016/j.it.2017.04.005, PMID: 28579324

[15] Wilmanski JM Petnicki-Ocwieja T Kobayashi KS. NLR proteins: integral members of innate immunity and mediators of inflammatory diseases. J Leukoc Biol. (2008) 83:13–30. doi: 10.1189/jlb.0607402, PMID: 17875812 PMC3256237

[16] Koonin EV Aravind L. The NACHT family – a new group of predicted NTPases implicated in apoptosis and MHC transcription activation. Trends Biochem Sci. (2000) 25:223–4. doi: 10.1016/S0968-0004(00)01577-2, PMID: 10782090

[17] Silke J Vucic D. Chapter two - IAP family of cell death and signaling regulators. In: Ashkenazi A Wells JA Yuan J, editors. Methods in enzymology. Lausanne, Switzerland: Academic Press (2014). p. 35–65., PMID: 10.1016/B978-0-12-801430-1.00002-0 25065885

[18] Howe K Schiffer PH Zielinski J Wiehe T Laird GK Marioni JC. Structure and evolutionary history of a large family of NLR proteins in the zebrafish. Open Biol. (2016) 6:160009. doi: 10.1098/rsob.160009, PMID: 27248802 PMC4852459

[19] Liu Y Zhang YB Liu TK Gui JF. Lineage-specific expansion of IFIT gene family: an insight into coevolution with IFN gene family. PloS One. (2013) 8:e66859. doi: 10.1371/journal.pone.0066859, PMID: 23818968 PMC3688568

[20] Bonardi V Cherkis K Nishimura MT Dangl JL. A new eye on NLR proteins: focused on clarity or diffused by complexity? . Curr Opin Immunol. (2012) 24:41–50. doi: 10.1016/j.coi.2011.12.006, PMID: 22305607 PMC3482489

[21] Tsankov BK Luchak A Carr C Philpott DJ. The effects of NOD-like receptors on adaptive immune responses. BioMed J. (2024) 47:100637. doi: 10.1016/j.bj.2023.100637, PMID: 37541620 PMC10796267

[22] Zhong Y Kinio A Saleh M. Functions of NOD-like receptors in human diseases. Front Immunol. (2013) 4:333. doi: 10.3389/fimmu.2013.00333, PMID: 24137163 PMC3797414

[23] Wain HM Lush MJ Ducluzeau F Khodiyar VK Povey S. Genew: the human gene nomenclature database, 2004 updates. Nucleic Acids Res. (2004) 32:D255–7. doi: 10.1093/nar/gkh072, PMID: 14681406 PMC308806

[24] Xu T Liao Z Su J. Pattern recognition receptors in grass carp Ctenopharyngodon idella: II. Organization and expression analysis of NOD-like receptors. Dev Comp Immunol. (2020) 110:103734. doi: 10.1016/j.dci.2020.103734, PMID: 32418892

[25] Laing KJ Purcell MK Winton JR Hansen JD. A genomic view of the NOD-like receptor family in teleost fish: identification of a novel NLR subfamily in zebrafish. BMC Evolutionary Biol. (2008) 8:42. doi: 10.1186/1471-2148-8-42, PMID: 18254971 PMC2268669

[26] Bi PY Killackey SA Schweizer L Girardin SE. NLRX1: Versatile functions of a mitochondrial NLR protein that controls mitophagy. BioMed J. (2024) 47:100635. doi: 10.1016/j.bj.2023.100635, PMID: 37574163 PMC10837482

[27] Chuphal B Rai U Roy B. Teleost NOD-like receptors and their downstream signaling pathways: A brief review. Fish Shellfish Immunol Rep. (2022) 3:100056. doi: 10.1016/j.fsirep.2022.100056, PMID: 36419601 PMC9680067

[28] Li J-Y Gao K Shao T Fan D-D Hu C-B Sun C-C. Characterization of an NLRP1 inflammasome from zebrafish reveals a unique sequential activation mechanism underlying inflammatory caspases in ancient vertebrates. J Immunol. (2018) 201:1946–66. doi: 10.4049/jimmunol.1800498, PMID: 30150286

[29] Rajendran KV Zhang J Liu S Kucuktas H Wang X Liu H. Pathogen recognition receptors in channel catfish: I. Identification, phylogeny and expression of NOD-like receptors. Dev Comp Immunol. (2012) 37:77–86. doi: 10.1016/j.dci.2011.12.005, PMID: 22200599

[30] Camacho C Coulouris G Avagyan V Ma N Papadopoulos J Bealer K. BLAST+: architecture and applications. BMC Bioinf. (2009) 10:421. doi: 10.1186/1471-2105-10-421, PMID: 20003500 PMC2803857

[31] Benson DA Cavanaugh M Clark K Karsch-Mizrachi I Ostell J Pruitt KD. GenBank. Nucleic Acids Res. (2017) 46:D41–7. doi: 10.1093/nar/gkw1070, PMID: 29140468 PMC5753231

[32] Altschul SF Madden TL Schäffer AA Zhang J Zhang Z Miller W. Gapped BLAST and PSI-BLAST: a new generation of protein database search programs. Nucleic Acids Res. (1997) 25:3389–402. doi: 10.1093/nar/25.17.3389, PMID: 9254694 PMC146917

[33] Altschul SF Gish W Miller W Myers EW Lipman DJ. Basic local alignment search tool. J Mol Biol. (1990) 215:403–10. doi: 10.1016/S0022-2836(05)80360-2, PMID: 2231712

[34] Saitou N Nei M. The neighbor-joining method: a new method for reconstructing phylogenetic trees. Mol Biol Evol. (1987) 4:406–25. doi: 10.1093/oxfordjournals.molbev.a040454, PMID: 3447015

[35] Kumar S Stecher G Li M Knyaz C Tamura K MEGA X. Molecular evolutionary genetics analysis across computing platforms. Mol Biol Evol. (2018) 35:1547–9. doi: 10.1093/molbev/msy096, PMID: 29722887 PMC5967553

[36] Schultz J Milpetz F Bork P Ponting CP. SMART, a simple modular architecture research tool: Identification of signaling domains. Proc Natl Acad Sci. (1998) 95:5857–64. doi: 10.1073/pnas.95.11.5857, PMID: 9600884 PMC34487

[37] Jumper J Evans R Pritzel A Green T Figurnov M Ronneberger O. Highly accurate protein structure prediction with AlphaFold. Nature. (2021) 596:583–9. doi: 10.1038/s41586-021-03819-2, PMID: 34265844 PMC8371605

[38] Varadi M Anyango S Deshpande M Nair S Natassia C Yordanova G. AlphaFold Protein Structure Database: massively expanding the structural coverage of protein-sequence space with high-accuracy models. Nucleic Acids Res. (2022) 50:D439–d444. doi: 10.1093/nar/gkab1061, PMID: 34791371 PMC8728224

[39] Steimle V Otten LA Zufferey M Mach B. Complementation cloning of an MHC class II transactivator mutated in hereditary MHC class II deficiency (or bare lymphocyte syndrome). Cell. (1993) 75:135–46. doi: 10.1016/S0092-8674(05)80090-X 8402893

[40] León MaChado JA Steimle V. The MHC class II transactivator CIITA: not (Quite) the odd-one-out anymore among NLR proteins. Int J Mol Sci. (2021) 33(3). doi: 10.3390/ijms22031074, PMID: 33499042 PMC7866136

[41] Spilianakis C Papamatheakis J Kretsovali A. Acetylation by PCAF enhances CIITA nuclear accumulation and transactivation of major histocompatibility complex class II genes. Mol Cell Biol. (2000) 20:8489–98. doi: 10.1128/MCB.20.22.8489-8498.2000, PMID: 11046145 PMC102155

[42] Cressman DE O'Connor WJ Greer SF Zhu XS Ting JP. Mechanisms of nuclear import and export that control the subcellular localization of class II transactivator. J Immunol. (2001) 167:3626–34. doi: 10.4049/jimmunol.167.7.3626, PMID: 11564775

[43] Raval A Weissman JD Howcroft TK Singer DS. The GTP-binding domain of class II transactivator regulates its nuclear export. J Immunol. (2003) 170:922–30. doi: 10.4049/jimmunol.170.2.922, PMID: 12517958

[44] Inohara N Koseki T Lin J del Peso L Lucas PC Chen FF. An induced proximity model for NF-&x3ba;B activation in the nod1/RICK and RIP signaling pathways. J Biol Chem. (2000) 275:27823–31. doi: 10.1074/jbc.M003415200, PMID: 10880512

[45] Bertin J Nir WJ Fischer CM Tayber OV Errada PR Grant JR. Human CARD4 protein is a novel CED-4/Apaf-1 cell death family member that activates NF-kappaB. J Biol Chem. (1999) 274:12955–8. doi: 10.1074/jbc.274.19.12955, PMID: 10224040

[46] Inohara N Koseki T del Peso L Hu Y Yee C Chen S. Nod1, an Apaf-1-like activator of caspase-9 and nuclear factor-kappaB. J Biol Chem. (1999) 274:14560–7. doi: 10.1074/jbc.274.21.14560, PMID: 10329646

[47] Zhu H Xiao C Chen J Guo B Wang W Tang Z. New insights into the structure domain and function of NLR family CARD domain containing 5. Cell Communication Signaling. (2025) 23:42. doi: 10.1186/s12964-024-02012-y, PMID: 39849460 PMC11755879

[48] Chou W-C Jha S Linhoff MW Ting JPY. The NLR gene family: from discovery to present day. Nat Rev Immunol. (2023) 23:635–54. doi: 10.1038/s41577-023-00849-x, PMID: 36973360 PMC11171412

[49] Ogura Y Bonen DK Inohara N Nicolae DL Chen FF Ramos R. A frameshift mutation in NOD2 associated with susceptibility to Crohn's disease. Nature. (2001) 411:603–6. doi: 10.1038/35079114, PMID: 11385577

[50] Hugot J-P Laurent-Puig P Gower-Rousseau C Olson JM Lee JC Beaugerie L. Mapping of a susceptibility locus for Crohn's disease on chromosome 16. Nature. (1996) 379:821–3. doi: 10.1038/379821a0, PMID: 8587604

[51] Ohmen JD Yang H-Y Yamamoto KK Zhao H-Y Ma Y Bentley LG. Susceptibility Locus for Inflammatory Bowel Disease on Chromosome 16 has a Role in Crohn's disease, but Not in Ulcerative Colitis. Hum Mol Genet. (1996) 5:1679–83. doi: 10.1093/hmg/5.10.1679, PMID: 8894707

[52] Curran ME Lau KF Hampe J Schreiber S Bridger S Macpherson AJS. Genetic analysis of inflammatory bowel disease in a large European cohort supports linkage to chromosomes 12 and 16. Gastroenterology. (1998) 115:1066–71. doi: 10.1016/S0016-5085(98)70075-7, PMID: 9797359

[53] Ogura Y Inohara N Benito A Chen FF Yamaoka S Núñez G. Nod2, a nod1/apaf-1 family member that is restricted to monocytes and activates NF-κB*. J Biol Chem. (2001) 276:4812–8. doi: 10.1074/jbc.M008072200, PMID: 11087742

[54] Girardin SE Boneca IG Viala J Chamaillard M Labigne A Thomas G. Nod2 is a general sensor of peptidoglycan through muramyl dipeptide (MDP) detection *. J Biol Chem. (2003) 278:8869–72. doi: 10.1074/jbc.C200651200, PMID: 12527755

[55] Swain B Basu M Sahoo BR Maiti NK Routray P Eknath AE. Molecular characterization of nucleotide binding and oligomerization domain (NOD)-2, analysis of its inductive expression and down-stream signaling following ligands exposure and bacterial infection in rohu (Labeo rohita). Dev Comp Immunol. (2012) 36:93–103. doi: 10.1016/j.dci.2011.06.018, PMID: 21767564

[56] Chamaillard M Hashimoto M Horie Y Masumoto J Qiu S Saab L. An essential role for NOD1 in host recognition of bacterial peptidoglycan containing diaminopimelic acid. Nat Immunol. (2003) 4:702–7. doi: 10.1038/ni945, PMID: 12796777

[57] Girardin SE Boneca IG Carneiro LA Antignac A Jéhanno M Viala J. Nod1 detects a unique muropeptide from gram-negative bacterial peptidoglycan. Science. (2003) 300:1584–7. doi: 10.1126/science.1084677, PMID: 12791997

[58] Poyet J-L Srinivasula SM Tnani M Razmara M Fernandes-Alnemri T Alnemri ES. Identification of ipaf, a human caspase-1-activating protein related to apaf-1. J Biol Chem. (2001) 276:28309–13. doi: 10.1074/jbc.C100250200, PMID: 11390368

[59] Zhang L Chen S Ruan J Wu J Tong AB Yin Q. Cryo-EM structure of the activated NAIP2-NLRC4 inflammasome reveals nucleated polymerization. Science. (2015) 350:404–9. doi: 10.1126/science.aac5789, PMID: 26449474 PMC4640189

[60] Martinon F Burns K Tschopp J. The inflammasome: A molecular platform triggering activation of inflammatory caspases and processing of proIL-β. Mol Cell. (2002) 10:417–26. doi: 10.1016/S1097-2765(02)00599-3, PMID: 12191486

[61] Alnemri ES Livingston DJ Nicholson DW Salvesen G Thornberry NA Wong WW. Human ICE/CED-3 protease nomenclature. Cell. (1996) 87:171. doi: 10.1016/S0092-8674(00)81334-3, PMID: 8861900

[62] Cerretti DP Kozlosky CJ Mosley B Nelson N Van Ness K Greenstreet TA. Molecular cloning of the interleukin-1 beta converting enzyme. Science. (1992) 256:97–100. doi: 10.1126/science.1373520, PMID: 1373520

[63] Thornberry NA Bull HG Calaycay JR Chapman KT Howard AD Kostura MJ. A novel heterodimeric cysteine protease is required for interleukin-1 beta processing in monocytes. Nature. (1992) 356:768–74. doi: 10.1038/356768a0, PMID: 1574116

[64] Stehlik C Lee SH Dorfleutner A Stassinopoulos A Sagara J Reed JC. Apoptosis-associated speck-like protein containing a caspase recruitment domain is a regulator of procaspase-1 activation. J Immunol. (2003) 171:6154–63. doi: 10.4049/jimmunol.171.11.6154, PMID: 14634131

[65] Moore CB Bergstralh DT Duncan JA Lei Y Morrison TE Zimmermann AG. NLRX1 is a regulator of mitochondrial antiviral immunity. Nature. (2008) 451:573–7. doi: 10.1038/nature06501, PMID: 18200010

[66] Seth RB Sun L Ea C-K Chen ZJ. Identification and characterization of MAVS, a mitochondrial antiviral signaling protein that activates NF-&x3ba;B and IRF3. Cell. (2005) 122:669–82. doi: 10.1016/j.cell.2005.08.012, PMID: 16125763

[67] Kawai T Takahashi K Sato S Coban C Kumar H Kato H. IPS-1, an adaptor triggering RIG-I- and Mda5-mediated type I interferon induction. Nat Immunol. (2005) 6:981–8. doi: 10.1038/ni1243, PMID: 16127453

[68] Xu L-G Wang Y-Y Han K-J Li L-Y Zhai Z Shu H-B. VISA is an adapter protein required for virus-triggered IFN-&x3b2; signaling. Mol Cell. (2005) 19:727–40. doi: 10.1016/j.molcel.2005.08.014, PMID: 16153868

[69] Meylan E Tschopp J. Toll-like receptors and RNA helicases: two parallel ways to trigger antiviral responses. Mol Cell. (2006) 22:561–9. doi: 10.1016/j.molcel.2006.05.012, PMID: 16762830

[70] Gitlin L Barchet W Gilfillan S Cella M Beutler B Flavell RA. Essential role of mda-5 in type I IFN responses to polyriboinosinic:polyribocytidylic acid and encephalomyocarditis picornavirus. Proc Natl Acad Sci. (2006) 103:8459–64. doi: 10.1073/pnas.0603082103, PMID: 16714379 PMC1464000

[71] Tattoli I Carneiro LA Jéhanno M Magalhaes JG Shu Y Philpott DJ. NLRX1 is a mitochondrial NOD-like receptor that amplifies NF-kappaB and JNK pathways by inducing reactive oxygen species production. EMBO Rep. (2008) 9:293–300. doi: 10.1038/sj.embor.7401161, PMID: 18219313 PMC2267388

[72] Cui J Zhu L Xia X Wang HY Legras X Hong J. NLRC5 negatively regulates the NF-&x3ba;B and type I interferon signaling pathways. Cell. (2010) 141:483–96. doi: 10.1016/j.cell.2010.03.040, PMID: 20434986 PMC3150216

[73] Meylan E Tschopp J Karin M. Intracellular pattern recognition receptors in the host response. Nature. (2006) 442:39–44. doi: 10.1038/nature04946, PMID: 16823444

[74] Chen ZJ. Ubiquitin signalling in the NF-kappaB pathway. Nat Cell Biol. (2005) 7:758–65. doi: 10.1038/ncb0805-758, PMID: 16056267 PMC1551980

[75] Meissner TB Li A Biswas A Lee K-H Liu Y-J Bayir E. NLR family member NLRC5 is a transcriptional regulator of MHC class I genes. Proc Natl Acad Sci. (2010) 107:13794–9. doi: 10.1073/pnas.1008684107, PMID: 20639463 PMC2922274

[76] Shastri N Cardinaud S Schwab SR Serwold T Kunisawa J. All the peptides that fit: the beginning, the middle, and the end of the MHC class I antigen-processing pathway. Immunol Rev. (2005) 207:31–41. doi: 10.1111/j.0105-2896.2005.00321.x, PMID: 16181325

[77] Reith W Mach B. The bare lymphocyte syndrome and the regulation of MHC expression. Annu Rev Immunol. (2001) 19:331–73. doi: 10.1146/annurev.immunol.19.1.331, PMID: 11244040

[78] van den Elsen PJ Gobin SJ van Eggermond MC Peijnenburg A. Regulation of MHC class I and II gene transcription: differences and similarities. Immunogenetics. (1998) 48:208–21. doi: 10.1007/s002510050425, PMID: 9683666

[79] van den Elsen PJ Peijnenburg A van Eggermond MC Gobin SJ. Shared regulatory elements in the promoters of MHC class I and class II genes. Immunol Today. (1998) 19:308–12. doi: 10.1016/S0167-5699(98)01287-0, PMID: 9666603

[80] Boss JM Jensen PE. Transcriptional regulation of the MHC class II antigen presentation pathway. Curr Opin Immunol. (2003) 15:105–11. doi: 10.1016/S0952-7915(02)00015-8, PMID: 12495741

[81] Martin BK Chin KC Olsen JC Skinner CA Dey A Ozato K. Induction of MHC class I expression by the MHC class II transactivator CIITA. Immunity. (1997) 6:591–600. doi: 10.1016/S1074-7613(00)80347-7, PMID: 9175837

[82] Ting JP Trowsdale J. Genetic control of MHC class II expression. Cell. (2002) 109 Suppl:S21–33. doi: 10.1016/S0092-8674(02)00696-7, PMID: 11983150

[83] Conti BJ Davis BK Zhang J O'Connor W Williams KL Ting JPY. CATERPILLER 16.2 (CLR16.2), a novel NBD/LRR family member that negatively regulates T cell function*. J Biol Chem. (2005) 280:18375–85. doi: 10.1074/jbc.M413169200, PMID: 15705585

[84] Li X Deng M Petrucelli AS Zhu C Mo J Zhang L. Viral DNA binding to NLRC3, an inhibitory nucleic acid sensor, unleashes STING, a cyclic dinucleotide receptor that activates type I interferon. Immunity. (2019) 50:591–599.e6. doi: 10.1016/j.immuni.2019.02.009, PMID: 30893587 PMC6469509

[85] Zhang L Mo J Swanson KV Wen H Petrucelli A Gregory SM. NLRC3, a member of the NLR family of proteins, is a negative regulator of innate immune signaling induced by the DNA sensor STING. Immunity. (2014) 40:329–41. doi: 10.1016/j.immuni.2014.01.010, PMID: 24560620 PMC4011014

[86] Hu S Du X Huang Y Fu Y Yang Y Zhan X. NLRC3 negatively regulates CD4+ T cells and impacts protective immunity during Mycobacterium tuberculosis infection. PloS Pathog. (2018) 14:e1007266. doi: 10.1371/journal.ppat.1007266, PMID: 30133544 PMC6122840

[87] Uchimura T Oyama Y Deng M Guo H Wilson JE Rampanelli E. The innate immune sensor NLRC3 acts as a rheostat that fine-tunes T cell responses in infection and autoimmunity. Immunity. (2018) 49:1049–1061.e6. doi: 10.1016/j.immuni.2018.10.008, PMID: 30566882 PMC6532657

[88] Wu H. Higher-order assemblies in a new paradigm of signal transduction. Cell. (2013) 153:287–92. doi: 10.1016/j.cell.2013.03.013, PMID: 23582320 PMC3687143

[89] Franklin BS Bossaller L De Nardo D Ratter JM Stutz A Engels G. The adaptor ASC has extracellular and 'prionoid' activities that propagate inflammation. Nat Immunol. (2014) 15:727–37. doi: 10.1038/ni.2913, PMID: 24952505 PMC4116676

[90] Cai X Chen J Xu H Liu S Jiang QX Halfmann R. Prion-like polymerization underlies signal transduction in antiviral immune defense and inflammasome activation. Cell. (2014) 156:1207–22. doi: 10.1016/j.cell.2014.01.063, PMID: 24630723 PMC4034535

[91] Baroja-Mazo A Martín-Sánchez F Gomez AI Martínez CM Amores-Iniesta J Compan V. The NLRP3 inflammasome is released as a particulate danger signal that amplifies the inflammatory response. Nat Immunol. (2014) 15:738–48. doi: 10.1038/ni.2919, PMID: 24952504

[92] Hu Z Yan C Liu P Huang Z Ma R Zhang C. Crystal structure of NLRC4 reveals its autoinhibition mechanism. Science. (2013) 341:172–5. doi: 10.1126/science.1236381, PMID: 23765277

[93] Sha Z Abernathy JW Wang S Li P Kucuktas H Liu H. NOD-like subfamily of the nucleotide-binding domain and leucine-rich repeat containing family receptors and their expression in channel catfish. Dev Comp Immunol. (2009) 33:991–9. doi: 10.1016/j.dci.2009.04.004, PMID: 19414032

[94] Swain B Basu M Samanta M. Molecular cloning and characterization of nucleotide binding and oligomerization domain-1 (NOD1) receptor in the Indian Major Carp, rohu (Labeo rohita), and analysis of its inductive expression and down-stream signalling molecules following ligands exposure and Gram-negative bacterial infections. Fish Shellfish Immunol. (2012) 32:899–908. doi: 10.1016/j.fsi.2012.02.018, PMID: 22530240

[95] Sahoo BR Swain B Dikhit MR Basu M Bej A Jayasankar P. Activation of Nucleotide-Binding Oligomerization Domain 1 (NOD1) Receptor Signaling in Labeo rohita by iE-DAP and Identification of Ligand-Binding Key Motifs in NOD1 by Molecular Modeling and Docking. Appl Biochem Biotechnol. (2013) 170:1282–309. doi: 10.1007/s12010-013-0263-6, PMID: 23657901

[96] Swain B Basu M Samanta M. NOD1 and NOD2 receptors in mrigal (Cirrhinus mrigala): Inductive expression and downstream signalling in ligand stimulation and bacterial infections. J Biosci. (2013) 38:533–48. doi: 10.1007/s12038-013-9330-y, PMID: 23938386

[97] Gao F-y Pang J-c Lu M-x Yang X-l Zhu H-p Ke X-l. Molecular characterization, expression and functional analysis of NOD1, NOD2 and NLRC3 in Nile tilapia (Oreochromis niloticus). Fish Shellfish Immunol. (2018) 73:207–19. doi: 10.1016/j.fsi.2017.12.012, PMID: 29242132

[98] Xie J Hodgkinson JW Katzenback BA Kovacevic N Belosevic M. Characterization of three Nod-like receptors and their role in antimicrobial responses of goldfish (Carassius auratus L.) macrophages to Aeromonas salmonicida and Mycobacterium marinum. Dev Comp Immunol. (2013) 39:180–7. doi: 10.1016/j.dci.2012.11.005, PMID: 23194927

[99] Li J Gao Y Xu T. Comparative genomic and evolution of vertebrate NOD1 and NOD2 genes and their immune response in miiuy croaker. Fish Shellfish Immunol. (2015) 46:387–97. doi: 10.1016/j.fsi.2015.06.026, PMID: 26108036

[100] Li S Chen X Hao G Geng X Zhan W Sun J. Identification and characterization of a novel NOD-like receptor family CARD domain containing 3 gene in response to extracellular ATP stimulation and its role in regulating LPS-induced innate immune response in Japanese flounder (Paralichthys olivaceus) head kidney macrophages. Fish Shellfish Immunol. (2016) 50:79–90. doi: 10.1016/j.fsi.2016.01.029, PMID: 26820104

[101] Zhao H Wang H Liu R Liang Y Li K Shan S. Activation of the NLRP1 inflammasome and its ligand recognition in the antibacterial immune response of common carp (Cyprinus carpio). Fish Shellfish Immunol. (2022) 125:238–46. doi: 10.1016/j.fsi.2022.05.019, PMID: 35588906

[102] Park HH Lo Y-C Lin S-C Wang L Yang JK Wu H. The death domain superfamily in intracellular signaling of apoptosis and inflammation. Annu Rev Immunol. (2007) 25:561–86. doi: 10.1146/annurev.immunol.25.022106.141656, PMID: 17201679 PMC2904440

[103] Bouchier-Hayes L Martin SJ. CARD games in apoptosis and immunity. EMBO Rep. (2002) 3:616–21. doi: 10.1093/embo-reports/kvf139, PMID: 12101092 PMC1084193

[104] Proell M Riedl SJ Fritz JH Rojas AM Schwarzenbacher R. The Nod-like receptor (NLR) family: a tale of similarities and differences. PloS One. (2008) 3:e2119. doi: 10.1371/journal.pone.0002119, PMID: 18446235 PMC2323615

[105] Qiao Q Yang C Zheng C Fontán L David L Yu X. Structural architecture of the CARMA1/Bcl10/MALT1 signalosome: nucleation-induced filamentous assembly. Mol Cell. (2013) 51:766–79. doi: 10.1016/j.molcel.2013.08.032, PMID: 24074955 PMC3929958

[106] Xu H He X Zheng H Huang LJ Hou F Yu Z. Structural basis for the prion-like MAVS filaments in antiviral innate immunity. eLife. (2014) 3:e01489. doi: 10.7554/eLife.01489, PMID: 24569476 PMC3932521

[107] Park HH. Caspase recruitment domains for protein interactions in cellular signaling (Review). Int J Mol Med. (2019) 43:1119–27. doi: 10.3892/ijmm.2019.4060, PMID: 30664151 PMC6365033

[108] Hofmann K Bucher P Tschopp J. The CARD domain: a new apoptotic signalling motif. Trends Biochem Sci. (1997) 22:155–6. doi: 10.1016/S0968-0004(97)01043-8, PMID: 9175472

[109] Damiano JS Reed JC. CARD proteins as therapeutic targets in cancer. Curr Drug Targets. (2004) 5:367–74. doi: 10.2174/1389450043345470, PMID: 15134219

[110] T.-h. Jang SH Jeong J-H Kim S Kim Y-G Park HH. Crystal structure of caspase recruiting domain (CARD) of apoptosis repressor with CARD (ARC) and its implication in inhibition of apoptosis. Sci Rep. (2015) 5:9847. doi: 10.1038/srep09847, PMID: 26038885 PMC4453921

[111] Ferrage F Dutta K Nistal-Villán E Patel JR Sánchez-Aparicio MT De Ioannes P. Structure and dynamics of the second CARD of human RIG-I provide mechanistic insights into regulation of RIG-I activation. Structure. (2012) 20:2048–61. doi: 10.1016/j.str.2012.09.003, PMID: 23063562 PMC3625992

[112] Mótyán JA Bagossi P Benkő S Tőzsér J. A molecular model of the full-length human NOD-like receptor family CARD domain containing 5 (NLRC5) protein. BMC Bioinf. (2013) 14:275. doi: 10.1186/1471-2105-14-275, PMID: 24044430 PMC3848420

[113] Sundaram B Tweedell RE Prasanth Kumar S Kanneganti T-D. The NLR family of innate immune and cell death sensors. Immunity. (2024) 57:674–99. doi: 10.1016/j.immuni.2024.03.012, PMID: 38599165 PMC11112261

[114] Hu Z Zhou Q Zhang C Fan S Cheng W Zhao Y. Structural and biochemical basis for induced self-propagation of NLRC4. Science. (2015) 350:399–404. doi: 10.1126/science.aac5489, PMID: 26449475

[115] Faustin B Lartigue L Bruey JM Luciano F Sergienko E Bailly-Maitre B. Reconstituted NALP1 inflammasome reveals two-step mechanism of caspase-1 activation. Mol Cell. (2007) 25:713–24. doi: 10.1016/j.molcel.2007.01.032, PMID: 17349957

[116] Bella J Hindle KL McEwan PA Lovell SC. The leucine-rich repeat structure. Cell Mol Life Sci. (2008) 65:2307–33. doi: 10.1007/s00018-008-8019-0, PMID: 18408889 PMC11131621

[117] Kobe B Kajava AV. When protein folding is simplified to protein coiling: the continuum of solenoid protein structures. Trends Biochem Sci. (2000) 25:509–15. doi: 10.1016/S0968-0004(00)01667-4, PMID: 11050437

[118] Kobe B Kajava AV. The leucine-rich repeat as a protein recognition motif. Curr Opin Struct Biol. (2001) 11:725–32. doi: 10.1016/S0959-440X(01)00266-4, PMID: 11751054

[119] Matsushima N Miyashita H Mikami T Kuroki Y. A nested leucine rich repeat (LRR) domain: The precursor of LRRs is a ten or eleven residue motif. BMC Microbiol. (2010) 10:235. doi: 10.1186/1471-2180-10-235, PMID: 20825685 PMC2946307

[120] Martin EC Spiridon L Goverse A Petrescu A-J. NLRexpress—A bundle of machine learning motif predictors—Reveals motif stability underlying plant Nod-like receptors diversity. Front Plant Sci Volume. (2022) 13. doi: 10.3389/fpls.2022.975888, PMID: 36186050 PMC9519389

[121] Kajava AV. Structural diversity of leucine-rich repeat proteins11Edited by F. Cohen. J Mol Biol. (1998) 277:519–27. doi: 10.1006/jmbi.1998.1643, PMID: 9533877

[122] Price SR Evans PR Nagai K. Crystal structure of the spliceosomal U2B"-U2A' protein complex bound to a fragment of U2 small nuclear RNA. Nature. (1998) 394:645–50. doi: 10.1038/29234, PMID: 9716128

[123] Seewald MJ Körner C Wittinghofer A Vetter IR. RanGAP mediates GTP hydrolysis without an arginine finger. Nature. (2002) 415:662–6. doi: 10.1038/415662a, PMID: 11832950

[124] Celikel R McClintock RA Roberts JR Mendolicchio GL Ware J Varughese KI. Modulation of alpha-thrombin function by distinct interactions with platelet glycoprotein Ibalpha. Science. (2003) 301:218–21. doi: 10.1126/science.1084183, PMID: 12855810

[125] Dumas JJ Kumar R Seehra J Somers WS Mosyak L. Crystal structure of the GpIbalpha-thrombin complex essential for platelet aggregation. Science. (2003) 301:222–6. doi: 10.1126/science.1083917, PMID: 12855811

[126] Kim JI Lee CJ Jin MS Lee CH Paik SG Lee H. Crystal structure of CD14 and its implications for lipopolysaccharide signaling. J Biol Chem. (2005) 280:11347–51. doi: 10.1074/jbc.M414607200, PMID: 15644310

[127] Jin MS Kim SE Heo JY Lee ME Kim HM Paik S-G. Crystal structure of the TLR1-TLR2 heterodimer induced by binding of a tri-acylated lipopeptide. Cell. (2007) 130:1071–82. doi: 10.1016/j.cell.2007.09.008, PMID: 17889651

[128] Franchi L Warner N Viani K Nuñez G. Function of Nod-like receptors in microbial recognition and host defense. Immunol Rev. (2009) 227:106–28. doi: 10.1111/j.1600-065X.2008.00734.x, PMID: 19120480 PMC2679989

[129] Tanabe T Chamaillard M Ogura Y Zhu L Qiu S Masumoto J. Regulatory regions and critical residues of NOD2 involved in muramyl dipeptide recognition. EMBO J. (2004) 23:1587–1597-1597. doi: 10.1038/sj.emboj.7600175, PMID: 15044951 PMC391079

[130] Hsu L-C Ali SR McGillivray S Tseng P-H Mariathasan S Humke EW. A NOD2–NALP1 complex mediates caspase-1-dependent IL-1β secretion in response to Bacillus anthracis infection and muramyl dipeptide. Proc Natl Acad Sci. (2008) 105:7803–8. doi: 10.1073/pnas.0802726105, PMID: 18511561 PMC2409384

[131] Kobe B Deisenhofer J. Crystal structure of porcine ribonuclease inhibitor. Protein leucine-rich repeats Nat. (1993) 366:751–6. doi: 10.1038/366751a0, PMID: 8264799

[132] Finger JN Lich JD Dare LC Cook MN Brown KK Duraiswami C. Autolytic proteolysis within the function to find domain (FIIND) is required for NLRP1 inflammasome activity*. J Biol Chem. (2012) 287:25030–7. doi: 10.1074/jbc.M112.378323, PMID: 22665479 PMC3408201

[133] Yu C-H Moecking J Geyer M Masters SL. Mechanisms of NLRP1-mediated autoinflammatory disease in humans and mice. J Mol Biol. (2018) 430:142–52. doi: 10.1016/j.jmb.2017.07.012, PMID: 28733143

[134] Zhu B Ouda R An N Tanaka T Kobayashi KS. The balance between nuclear import and export of NLRC5 regulates MHC class I transactivation. J Biol Chem. (2024) 300:107205. doi: 10.1016/j.jbc.2024.107205, PMID: 38519032 PMC11044055

[135] Hasegawa M Fujimoto Y Lucas PC Nakano H Fukase K Núñez G. A critical role of RICK/RIP2 polyubiquitination in Nod-induced NF-κB activation. EMBO J. (2008) 27:373–383-383. doi: 10.1038/sj.emboj.7601962, PMID: 18079694 PMC2234345

[136] Coutermarsh-Ott S Eden K Allen IC. Beyond the inflammasome: regulatory NOD-like receptor modulation of the host immune response following virus exposure. J Gen Virol. (2016) 97:825–38. doi: 10.1099/jgv.0.000401, PMID: 26763980 PMC4854363

[137] Chang MX Xiong F Wu XM Hu YW. The expanding and function of NLRC3 or NLRC3-like in teleost fish: Recent advances and novel insights. Dev Comp Immunol. (2021) 114:103859. doi: 10.1016/j.dci.2020.103859, PMID: 32896535

[138] Li Q Chen Y Wang P Sun Y Xu T. PSMD13 inhibits NF-κB pathway by targeting TAK1 for K63-linked ubiquitination in miiuy croaker (Miichthys miiuy). Fish Shellfish Immunol. (2023) 138:108857. doi: 10.1016/j.fsi.2023.108857, PMID: 37257570

[139] Bao S-Y Sun Q-X Yao C-L. The interaction of TAK1 and TAB1 enhances LPS-induced cytokine release via modulating NF-κB activation (Larimichthys crocea). Fish Shellfish Immunol. (2018) 74:450–8. doi: 10.1016/j.fsi.2018.01.005, PMID: 29325713

[140] Zhao F Li Y-W Pan H-J Shi C-B Luo X-C Li A-X. TAK1-binding proteins (TAB1 and TAB2) in grass carp (Ctenopharyngodon idella): Identification, characterization, and expression analysis after infection with Ichthyophthirius multifiliis. Fish Shellfish Immunol. (2014) 38:389–99. doi: 10.1016/j.fsi.2014.04.008, PMID: 24747054

[141] Correa RG Tergaonkar V Ng JK Dubova I Izpisua-Belmonte JC Verma IM. Characterization of NF-kappa B/I kappa B proteins in zebra fish and their involvement in notochord development. Mol Cell Biol. (2004) 24:5257–68. doi: 10.1128/MCB.24.12.5257-5268.2004, PMID: 15169890 PMC419862

[142] Verma IM Stevenson JK Schwarz EM Van Antwerp D Miyamoto S. Rel/NF-kappa B/I kappa B family: intimate tales of association and dissociation. Genes Dev. (1995) 9:2723–35. doi: 10.1101/gad.9.22.2723, PMID: 7590248

[143] Bi D Gao Y Chu Q Cui J Xu T. NOD1 is the innate immune receptor for iE-DAP and can activate NF-κB pathway in teleost fish. Dev Comp Immunol. (2017) 76:238–46. doi: 10.1016/j.dci.2017.06.012, PMID: 28655577

[144] Ren Y Liu SF Nie L Cai SY Chen J. Involvement of ayu NOD2 in NF-κB and MAPK signaling pathways: Insights into functional conservation of NOD2 in antibacterial innate immunity. Zool Res. (2019) 40:77–88. doi: 10.24272/j.issn.2095-8137.2018.066, PMID: 29872030 PMC6378557

[145] Tang P Chen Y Chen D Zhu H Dai S Zhou J. Transcriptome analysis reveals the mechanism of cortisol through GR regulating the expression of inflammatory cytokines in Siberian sturgeon (Acipenser baerii) after LPS treatment *in vitro* . Fish Shellfish Immunol. (2025) 161:110262. doi: 10.1016/j.fsi.2025.110262, PMID: 40058676

[146] Wang Q Li M Hu G Xiao G Teng S. Characterization of a novel activating protein-1 (AP-1) gene and the association of its single nucleotide polymorphisms with vibrio resistance in Tegillarca granosa. Fish Shellfish Immunol. (2022) 124:552–62. doi: 10.1016/j.fsi.2022.04.023, PMID: 35489594

[147] Le Bourhis L Benko S Girardin SE. Nod1 and Nod2 in innate immunity and human inflammatory disorders. Biochem Soc Trans 35(Pt. (2007) 6):1479–84. doi: 10.1042/BST0351479, PMID: 18031249

[148] Rosenzweig HL Kawaguchi T Martin TM Planck SR Davey MP Rosenbaum JT. Nucleotide oligomerization domain-2 (NOD2)-induced uveitis: dependence on IFN-gamma. Invest Ophthalmol Vis Sci. (2009) 50:1739–45. doi: 10.1167/iovs.08-2756, PMID: 19098321 PMC3089593

[149] Wu XM Zhang J Li PW Hu YW Cao L Ouyang S. NOD1 promotes antiviral signaling by binding viral RNA and regulating the interaction of MDA5 and MAVS. J Immunol. (2020) 204:2216–31. doi: 10.4049/jimmunol.1900667, PMID: 32169843

[150] Sabbah A Chang TH Harnack R Frohlich V Tominaga K Dube PH. Activation of innate immune antiviral responses by Nod2. Nat Immunol. (2009) 10:1073–80. doi: 10.1038/ni.1782, PMID: 19701189 PMC2752345

[151] Fan Y-H Roy S Mukhopadhyay R Kapoor A Duggal P Wojcik GL. Role of nucleotide-binding oligomerization domain 1 (NOD1) and its variants in human cytomegalovirus control *in vitro* and *in vivo* . Proc Natl Acad Sci. (2016) 113:E7818–27. doi: 10.1073/pnas.1611711113, PMID: 27856764 PMC5137695

[152] Lecat A Piette J Legrand-Poels S. The protein Nod2: An innate receptor more complex than previously assumed. Biochem Pharmacol. (2010) 80:2021–31. doi: 10.1016/j.bcp.2010.07.016, PMID: 20643110

[153] Zou PF Chang MX Li Y Xue NN Li JH Chen SN. NOD2 in zebrafish functions in antibacterial and also antiviral responses via NF-κB, and also MDA5, RIG-I and MAVS. Fish Shellfish Immunol. (2016) 55:173–85. doi: 10.1016/j.fsi.2016.05.031, PMID: 27235368

[154] Zou PF Chang MX Li Y Huan Zhang S Fu JP Chen SN. Higher antiviral response of RIG-I through enhancing RIG-I/MAVS-mediated signaling by its long insertion variant in zebrafish. Fish Shellfish Immunol. (2015) 43:13–24. doi: 10.1016/j.fsi.2014.12.001, PMID: 25524497

[155] Zou PF Chang MX Xue NN Liu XQ Li JH Fu JP. Melanoma differentiation-associated gene 5 in zebrafish provoking higher interferon-promoter activity through signalling enhancing of its shorter splicing variant. Immunology. (2014) 141:192–202. doi: 10.1111/imm.12179, PMID: 24116956 PMC3904240

[156] Biacchesi S LeBerre M Lamoureux A Louise Y Lauret E Boudinot P. Mitochondrial antiviral signaling protein plays a major role in induction of the fish innate immune response against RNA and DNA viruses. J Virol. (2009) 83:7815–27. doi: 10.1128/JVI.00404-09, PMID: 19474100 PMC2715792

[157] Chang M Collet B Nie P Lester K Campbell S Secombes Christopher J. Expression and functional characterization of the RIG-I-like receptors MDA5 and LGP2 in rainbow trout (Oncorhynchus mykiss). J Virol. (2011) 85:8403–12. doi: 10.1128/JVI.00445-10, PMID: 21680521 PMC3147945

[158] Beutler B Eidenschenk C Crozat K Imler J-L Takeuchi O Hoffmann JA. Genetic analysis of resistance to viral infection. Nat Rev Immunol. (2007) 7:753–66. doi: 10.1038/nri2174, PMID: 17893693

[159] Shibasaki Y Hatanaka C Matsuura Y Miyazawa R Yabu T Moritomo T. Effects of IFNγ administration on allograft rejection in ginbuna crucian carp. Dev Comp Immunol. (2016) 62:108–15. doi: 10.1016/j.dci.2016.04.021, PMID: 27156851

[160] Gan Z Chen SN Huang B Zou J Nie P Fish type I. and type II interferons: composition, receptor usage, production and function. Rev Aquaculture. (2020) 12:773–804. doi: 10.1111/raq.12349

[161] Zou J Secombes CJ. The function of fish cytokines. Biol (Basel). (2016) 5(2):23. doi: 10.3390/biology5020023, PMID: 27231948 PMC4929537

[162] Yin L Lv M Qiu X Wang X Zhang A Yang K. IFN-γ Manipulates NOD1-mediated interaction of autophagy and edwardsiella piscicida to augment intracellular clearance in fish. J Immunol. (2021) 207:1087–98. doi: 10.4049/jimmunol.2100151, PMID: 34341174

[163] Li Z Shang D. NOD1 and NOD2: essential monitoring partners in the innate immune system. Curr Issues Mol Biol. (2024) 46:9463–79. doi: 10.3390/cimb46090561, PMID: 39329913 PMC11430502

[164] Travassos LH Carneiro LAM Ramjeet M Hussey S Kim Y-G Magalhães JG. Nod1 and Nod2 direct autophagy by recruiting ATG16L1 to the plasma membrane at the site of bacterial entry. Nat Immunol. (2010) 11:55–62. doi: 10.1038/ni.1823, PMID: 19898471

[165] Lipinski S Till A Sina C Arlt A Grasberger H Schreiber S. DUOX2-derived reactive oxygen species are effectors of NOD2-mediated antibacterial responses. J Cell Sci 122(Pt. (2009) 19):3522–30. doi: 10.1242/jcs.050690, PMID: 19759286

[166] Flores MV Crawford KC Pullin LM Hall CJ Crosier KE Crosier PS. Dual oxidase in the intestinal epithelium of zebrafish larvae has anti-bacterial properties. Biochem Biophys Res Commun. (2010) 400:164–8. doi: 10.1016/j.bbrc.2010.08.037, PMID: 20709024

[167] Allen IC. A NOD to zebrafish models of inflammatory bowel disease pathogenesis. Dis Model Mech. (2011) 4:711–2. doi: 10.1242/dmm.008805, PMID: 22065838 PMC3209637

[168] Sun D Xu J Zhang W Song C Gao C He Y. Negative regulator NLRC3: Its potential role and regulatory mechanism in immune response and immune-related diseases. Front Immunol. (2022) 13:1012459. doi: 10.3389/fimmu.2022.1012459, PMID: 36341336 PMC9630602

[169] Xu YQ Li Y Han KH Zhang JX Li KQ Jiang J. NLRC3 regulates RIP2, STING, TBK1, and TRAF6 mediated type I IFN signaling and inflammatory response in large yellow croaker Larimichthys crocea. Fish Shellfish Immunol. (2025) 162:110351. doi: 10.1016/j.fsi.2025.110351, PMID: 40252745

[170] Zhang L Chen H Zhao X Chen Y Li S Xiao T. NLRC3 attenuates antiviral innate immune response by targeting IRF7 in grass carp (Ctenopharyngodon idelus). Int J Mol Sci. (2025) 26(2):840. doi: 10.3390/ijms26020840, PMID: 39859554 PMC11766192

[171] Fang H Wu XM Hu YW Song YJ Zhang J Chang MX. NLRC3-like 1 inhibits NOD1-RIPK2 pathway via targeting RIPK2. Dev Comp Immunol. (2020) 112:103769. doi: 10.1016/j.dci.2020.103769, PMID: 32634524

[172] Xie J Belosevic M. Characterization and functional assessment of the NLRC3-like molecule of the goldfish (Carassius auratus L. ). Dev Comp Immunol. (2018) 79:1–10. doi: 10.1016/j.dci.2017.09.021, PMID: 28988993

[173] Hu YW Wu XM Ren SS Cao L Nie P Chang MX. NOD1 deficiency impairs CD44a/Lck as well as PI3K/Akt pathway. Sci Rep. (2017) 7:2979. doi: 10.1038/s41598-017-03258-y, PMID: 28592872 PMC5462776

[174] Wu XM Chen WQ Hu YW Cao L Nie P Chang MX. RIP2 is a critical regulator for NLRs signaling and MHC antigen presentation but not for MAPK and PI3K/akt pathways. Front Immunol Volume. (2018) 9. doi: 10.3389/fimmu.2018.00726, PMID: 29692779 PMC5903030

[175] Wu XM Hu YW Xue NN Ren SS Chen SN Nie P. Role of zebrafish NLRC5 in antiviral response and transcriptional regulation of MHC related genes. Dev Comp Immunol. (2017) 68:58–68. doi: 10.1016/j.dci.2016.11.018, PMID: 27876605

[176] Kuenzel S Till A Winkler M Häsler R Lipinski S Jung S. The nucleotide-binding oligomerization domain-like receptor NLRC5 is involved in IFN-dependent antiviral immune responses. J Immunol. (2010) 184:1990–2000. doi: 10.4049/jimmunol.0900557, PMID: 20061403

[177] Benkő S Kovács EG Hezel F Kufer TA. NLRC5 functions beyond MHC I regulation—What do we know so far? Front Immunol Volume. (2017) 8:2017. doi: 10.3389/fimmu.2017.00150, PMID: 28261210 PMC5313500

[178] Ludigs K Seguín-Estévez Q Lemeille S Ferrero I Rota G Chelbi S. NLRC5 exclusively transactivates MHC class I and related genes through a distinctive SXY module. PloS Genet. (2015) 11:e1005088. doi: 10.1371/journal.pgen.1005088, PMID: 25811463 PMC4374748

[179] Yoshihama S Roszik J Downs I Meissner TB Vijayan S Chapuy B. NLRC5/MHC class I transactivator is a target for immune evasion in cancer. Proc Natl Acad Sci. (2016) 113:5999–6004. doi: 10.1073/pnas.1602069113, PMID: 27162338 PMC4889388

[180] Benko S Magalhaes JG Philpott DJ Girardin SE. NLRC5 limits the activation of inflammatory pathways. J Immunol. (2010) 185:1681–91. doi: 10.4049/jimmunol.0903900, PMID: 20610642

[181] Staehli F Ludigs K Heinz LX Seguín-Estévez Q Ferrero I Braun M. NLRC5 deficiency selectively impairs MHC class I- dependent lymphocyte killing by cytotoxic T cells. J Immunol. (2012) 188:3820–8. doi: 10.4049/jimmunol.1102671, PMID: 22412192

[182] Neerincx A Rodriguez GM Steimle V Kufer TA. NLRC5 controls basal MHC class I gene expression in an MHC enhanceosome-dependent manner. J Immunol. (2012) 188:4940–50. doi: 10.4049/jimmunol.1103136, PMID: 22490867

[183] Wang B Lan X Lin S Xu H Zhang X Yin J. NLRX1 in fish: A negative regulator of innate immunity during Edwardsiella piscicida infection via targeting TRAF6 through the NACHT domain. Aquaculture. (2025) 594:741464. doi: 10.1016/j.aquaculture.2024.741464

[184] Fang R Jiang Q Zhou X Wang C Guan Y Tao J. MAVS activates TBK1 and IKKϵ through TRAFs in NEMO dependent and independent manner. PloS Pathog. (2017) 13:e1006720. doi: 10.1371/journal.ppat.1006720, PMID: 29125880 PMC5699845

[185] Cao Y Chen Z Huang J Wu H Zou J Feng H. Black carp TUFM collaborates with NLRX1 to inhibit MAVS-mediated antiviral signaling pathway. Dev Comp Immunol. (2021) 122:104134. doi: 10.1016/j.dci.2021.104134, PMID: 34000319

[186] Song X Li W Xie X Zou Z Wei J Wu H. NLRX1 of black carp suppresses MAVS-mediated antiviral signaling through its NACHT domain. Dev Comp Immunol. (2019) 96:68–77. doi: 10.1016/j.dci.2019.03.001, PMID: 30853538

[187] Ratsimandresy RA Dorfleutner A Stehlik C. An update on PYRIN domain-containing pattern recognition receptors: from immunity to pathology. Front Immunol. (2013) 4:440. doi: 10.3389/fimmu.2013.00440, PMID: 24367371 PMC3856626

[188] Srinivasula SM Poyet J-L Razmara M Datta P Zhang Z Alnemri ES. The PYRIN-CARD protein ASC is an activating adaptor for caspase-1*. J Biol Chem. (2002) 277:21119–22. doi: 10.1074/jbc.C200179200, PMID: 11967258

[189] Stehlik C Lee SH Dorfleutner A Stassinopoulos A Sagara J Reed JC. Apoptosis-associated speck-like protein containing a caspase recruitment domain is a regulator of procaspase-1 activation 1. J Immunol. (2003) 171:6154–63. doi: 10.4049/jimmunol.171.11.6154, PMID: 14634131

[190] Hasegawa M Imamura R Kinoshita T Matsumoto N Masumoto J Inohara N. ASC-mediated NF-κB activation leading to interleukin-8 production requires caspase-8 and is inhibited by CLARP*. J Biol Chem. (2005) 280:15122–30. doi: 10.1074/jbc.M412284200, PMID: 15701651

[191] Pierini R Juruj C Perret M Jones CL Mangeot P Weiss DS. AIM2/ASC triggers caspase-8-dependent apoptosis in Francisella-infected caspase-1-deficient macrophages. Cell Death Differ. (2012) 19:1709–21. doi: 10.1038/cdd.2012.51, PMID: 22555457 PMC3438500

[192] D'Osualdo A Weichenberger CX Wagner RN Godzik A Wooley J Reed JC. CARD8 and NLRP1 undergo autoproteolytic processing through a ZU5-like domain. PloS One. (2011) 6:e27396. doi: 10.1371/journal.pone.0027396, PMID: 22087307 PMC3210808

[193] Chang MX. Emerging mechanisms and functions of inflammasome complexes in teleost fish. Front Immunol Volume. (2023) 14:2023. doi: 10.3389/fimmu.2023.1065181, PMID: 36875130 PMC9978379

[194] Broz P Dixit VM. Inflammasomes: mechanism of assembly. Regul signalling Nat Rev Immunol. (2016) 16:407–20. doi: 10.1038/nri.2016.58, PMID: 27291964

[195] Lamkanfi M Vishva M. Dixit, mechanisms and functions of inflammasomes. Cell. (2014) 157:1013–22. doi: 10.1016/j.cell.2014.04.007, PMID: 24855941

[196] Downs KP Nguyen H Dorfleutner A Stehlik C. An overview of the non-canonical inflammasome. Mol Aspects Med. (2020) 76:100924. doi: 10.1016/j.mam.2020.100924, PMID: 33187725 PMC7808250

[197] Boucher D Monteleone M Coll RC Chen KW Ross CM Teo JL. Caspase-1 self-cleavage is an intrinsic mechanism to terminate inflammasome activity. J Exp Med. (2018) 215:827–40. doi: 10.1084/jem.20172222, PMID: 29432122 PMC5839769

[198] Chavarría-Smith J Vance RE. The NLRP1 inflammasomes. Immunol Rev. (2015) 265:22–34. doi: 10.1111/imr.12283, PMID: 25879281

[199] Li JY Wang YY Shao T Fan DD Lin AF Xiang LX. The zebrafish NLRP3 inflammasome has functional roles in ASC-dependent interleukin-1β maturation and gasdermin E-mediated pyroptosis. J Biol Chem. (2020) 295:1120–41. doi: 10.1016/S0021-9258(17)49920-0, PMID: 31852739 PMC6983845

[200] Zhao Y Liang Y Chen Q Shan S Yang G Li H. The function of NLRP3 in anti-infection immunity and inflammasome assembly of common carp (Cyprinus carpio L. ). Fish Shellfish Immunol. (2024) 145:109367. doi: 10.1016/j.fsi.2024.109367, PMID: 38211703

[201] Gorfu G Cirelli Kimberly M Melo Mariane B Mayer-Barber K Crown D Koller Beverly H. Dual role for inflammasome sensors NLRP1 and NLRP3 in murine resistance to toxoplasma gondii. mBio. (2014) 5:e01117-13. doi: 10.1128/mbio.01117-13, PMID: 24549849 PMC3944820

[202] Acevedo W Morán-Figueroa R Vargas-Chacoff L Morera FJ Pontigo JP. Revealing the salmo salar NLRP3 inflammasome: insights from structural modeling and transcriptome analysis. Int J Mol Sci. (2023) 24(19):14556. doi: 10.3390/ijms241914556, PMID: 37834004 PMC10572965

[203] Oehlers SH Flores MV Hall CJ Swift S Crosier KE Crosier PS. The inflammatory bowel disease (IBD) susceptibility genes NOD1 and NOD2 have conserved anti-bacterial roles in zebrafish. Dis Models Mech. (2011) 4:832–41. doi: 10.1242/dmm.006122, PMID: 21729873 PMC3209652

[204] Bi D Wang Y Gao Y Li X Chu Q Cui J. Recognition of lipopolysaccharide and activation of NF-κB by cytosolic sensor NOD1 in teleost fish. Front Immunol. (2018) 9:1413. doi: 10.3389/fimmu.2018.01413, PMID: 30013548 PMC6036275

[205] Swain B Campodonico VA Curtiss R. Recombinant Attenuated Edwardsiella piscicida Vaccine Displaying Regulated Lysis to Confer Biological Containment and Protect Catfish against Edwardsiellosis. Vaccines. (2023) 11:1470. doi: 10.3390/vaccines11091470, PMID: 37766146 PMC10534663

[206] Li M Wang Q-l Lu Y Chen S-l Li Q Sha Z-x. Expression profiles of NODs in channel catfish (Ictalurus punctatus) after infection with Edwardsiella tarda, Aeromonas hydrophila, Streptococcus iniae and channel catfish hemorrhage reovirus. Fish Shellfish Immunol. (2012) 33:1033–41. doi: 10.1016/j.fsi.2012.06.033, PMID: 22796486

[207] Huang S Huang Y Su T Huang R Su L Wu Y. Orange-spotted grouper nervous necrosis virus-encoded protein A induces interferon expression via RIG-I/MDA5-MAVS-TBK1-IRF3 signaling in fish cells. Microbiol Spectr. (2024) 12:e0453222. doi: 10.1128/spectrum.04532-22, PMID: 38095472 PMC10783131

[208] Rao Y Su J. Insights into the Antiviral Immunity against Grass Carp (Ctenopharyngodon idella) Reovirus (GCRV) in Grass Carp. J Immunol Res. (2015) 2015:670437. doi: 10.1155/2015/670437, PMID: 25759845 PMC4337036

[209] Chen L Li Q Su J Yang C Li Y Rao Y. Trunk kidney of grass carp (Ctenopharyngodon idella) mediates immune responses against GCRV and viral/bacterial PAMPs *in vivo* and *in vitro* . Fish Shellfish Immunol. (2013) 34:909–19. doi: 10.1016/j.fsi.2013.01.003, PMID: 23333439

[210] Chen WQ Xu QQ Chang MX Nie P Peng KM. Molecular characterization and expression analysis of nuclear oligomerization domain proteins NOD1 and NOD2 in grass carp Ctenopharyngodon idella. Fish Shellfish Immunol. (2010) 28:18–29. doi: 10.1016/j.fsi.2009.09.012, PMID: 19766192

[211] Xiao F Liao L Xu Q He Z Xiao T Wang J. Host-microbiota interactions and responses to grass carp reovirus infection in Ctenopharyngodon idellus. Environ Microbiol. (2021) 23:431–47. doi: 10.1111/1462-2920.15330, PMID: 33201573

[212] Pan J-M Liang Y Zhu K-C Guo H-Y Liu B-S Zhang N. Identification of the NOD-like receptor family of golden pompano and expression in response to bacterial and parasitic exposure reveal its key role in innate immunity. Dev Comp Immunol. (2024) 152:105123. doi: 10.1016/j.dci.2023.105123, PMID: 38135022

[213] Maharana J Swain B Sahoo BR Dikhit MR Basu M Mahapatra AS. Identification of MDP (muramyl dipeptide)-binding key domains in NOD2 (nucleotide-binding and oligomerization domain-2) receptor of Labeo rohita. Fish Physiol Biochem. (2013) 39:1007–23. doi: 10.1007/s10695-012-9758-2, PMID: 23255217

[214] Yuan S Sun Z Gao Q Li Z Qi Z Zheng S. Molecular characterization and expression analysis of NLRC3-like, ASC, and caspase1 in spotted sea bass (Lateolabrax maculatus). Fishes. (2023) 8:378. doi: 10.3390/fishes8070378

[215] Unajak S Santos MD J.-i. Hikima T-S Kondo H Hirono I Aoki T. Molecular characterization, expression and functional analysis of a nuclear oligomerization domain proteins subfamily C (NLRC) in Japanese flounder (Paralichthys olivaceus). Fish Shellfish Immunol. (2011) 31:202–11. doi: 10.1016/j.fsi.2011.05.007, PMID: 21642003

[216] Tang X Zhang Y Xing J Sheng X Chi H Zhan W. Proteomic and Phosphoproteomic Analysis Reveals Differential Immune Response to Hirame Novirhabdovirus (HIRRV) Infection in the Flounder (Paralichthys olivaceus) under Different Temperature. Biol (Basel). (2023) 12(8):1145. doi: 10.3390/biology12081145, PMID: 37627029 PMC10452491

[217] Zhao L Huang J Li Y Wu S Kang Y. Skin immune response of rainbow trout (Oncorhynchus mykiss) infected with infectious hematopoietic necrosis virus. Aquaculture Int. (2023) 31:3275–95. doi: 10.1007/s10499-023-01122-7

[218] Gervais O Papadopoulou A Gratacap R Hillestad B Tinch AE Martin SAM. Transcriptomic response to ISAV infection in the gills, head kidney and spleen of resistant and susceptible Atlantic salmon. BMC Genomics. (2022) 23:775. doi: 10.1186/s12864-022-09007-4, PMID: 36443659 PMC9703674

[219] Tsoulia T Sundaram AYM Braaen S Jørgensen JB Rimstad E Wessel Ø. Transcriptomics of early responses to purified Piscine orthoreovirus-1 in Atlantic salmon (Salmo salar L. ) red Blood Cells compared to non-susceptible Cell lines. Front Immunol Volume. (2024) 15:2024. doi: 10.3389/fimmu.2024.1359552, PMID: 38420125 PMC10899339

[220] Chen F Zhang W Xu X Gui L Lin Y Wu M. Identification of genes related to resistance to ichthyophthirius multifiliis based on co-expression network analysis in grass carp. Mar Biotechnol. (2023) 25:824–36. doi: 10.1007/s10126-023-10243-2, PMID: 37610535

[221] Pontigo JP Agüero MJ Sánchez P Oyarzún R Vargas-Lagos C Mancilla J. Identification and expressional analysis of NLRC5 inflammasome gene in smolting Atlantic salmon (Salmo salar). Fish Shellfish Immunol. (2016) 58:259–65. doi: 10.1016/j.fsi.2016.09.031, PMID: 27640334

[222] Talbot A Gargan L Moran G Prudent L O’Connor I Mirimin L. Investigation of the transcriptomic response in Atlantic salmon (Salmo salar) gill exposed to Paramoeba Perurans during early onset of disease. Sci Rep. (2021) 11:20682. doi: 10.1038/s41598-021-99996-1, PMID: 34667245 PMC8526816

[223] Sood N Verma DK Paria A Yadav SC Yadav MK Bedekar MK. Transcriptome analysis of liver elucidates key immune-related pathways in Nile tilapia Oreochromis niloticus following infection with tilapia lake virus. Fish Shellfish Immunol. (2021) 111:208–19. doi: 10.1016/j.fsi.2021.02.005, PMID: 33577877

[224] Zheng D Liwinski T Elinav E. Inflammasome activation and regulation: toward a better understanding of complex mechanisms. Cell Discov. (2020) 6:36. doi: 10.1038/s41421-020-0167-x, PMID: 32550001 PMC7280307

[225] Dahle MK Jørgensen JB. Antiviral defense in salmonids – Mission made possible? . Fish Shellfish Immunol. (2019) 87:421–37. doi: 10.1016/j.fsi.2019.01.043, PMID: 30708056

[226] Timmerhaus G Krasnov A Takle H Afanasyev S Nilsen P Rode M. Comparison of Atlantic salmon individuals with different outcomes of cardiomyopathy syndrome (CMS). BMC Genomics. (2012) 13:205. doi: 10.1186/1471-2164-13-205, PMID: 22646522 PMC3443006

[227] Xu W Li M Wang S Song Y Xu H Lin K. Transcriptomic profiles of spotted knifejaw (Oplegnathus punctatus) spleen in response to Megalocytivirus infection. Aquaculture. (2022) 555:738212. doi: 10.1016/j.aquaculture.2022.738212

[228] Song Y Wang L Li K Zhang M Chen S. Molecular identification and expression analysis of NOD1/2 and TBK1 in response to viral or bacterial infection in the spotted knifejaw (Oplegnathus punctatus). Anim (Basel). (2025) 15(7):1006. doi: 10.3390/ani15071006, PMID: 40218399 PMC11987823

[229] Liao J Kang S Zhang L Zhang D Xu Z Qin Q. Isolation and identification of a megalocytivirus strain (SKIV-TJ) from cultured spotted knifejaw (Oplegnathus punctatus) in China and its pathogenicity analysis. Fish Shellfish Immunol. (2023) 141:109034. doi: 10.1016/j.fsi.2023.109034, PMID: 37640124

[230] Rajendran KV Zhang J Liu S Peatman E Kucuktas H Wang X. Pathogen recognition receptors in channel catfish: II. Identification, phylogeny and expression of retinoic acid-inducible gene I (RIG-I)-like receptors (RLRs). Dev Comp Immunol. (2012) 37:381–9. doi: 10.1016/j.dci.2012.02.004, PMID: 22387588

[231] Xia Y Yu X Yuan Z Yang Y Liu Y. Whole-Transcriptome Analysis Reveals Potential CeRNA Regulatory Mechanism in Takifugu rubripes against Cryptocaryon irritans Infection. Biol (Basel). (2024) 13:788. doi: 10.3390/biology13100788, PMID: 39452097 PMC11504436

[232] Li Z Jiang B Zhong Z Cao J Li H Wang C. Skin transcriptomic analysis and immune-related gene expression of golden pompano (Trachinotus ovatus) after Amyloodinium ocellatum infection. Fish Shellfish Immunol. (2022) 128:188–95. doi: 10.1016/j.fsi.2022.07.052, PMID: 35870749

[233] Mladineo I Hrabar J. Seventy years of coexistence: Parasites and Mediterranean fish aquaculture. Fish Shellfish Immunol. (2025) 162:110355. doi: 10.1016/j.fsi.2025.110355, PMID: 40254086

[234] Maurelli AT Blackmon B Curtiss R. Temperature-dependent expression of virulence genes in Shigella species. Infection Immun. (1984) 43:195–201. doi: 10.1128/iai.43.1.195-201.1984, PMID: 6360895 PMC263409

[235] Wang JQ Liu YR Xia Q Chen RN Liang J Xia QR. Emerging roles for NLRC5 in immune diseases. Front Pharmacol. (2019) 10:1352. doi: 10.3389/fphar.2019.01352, PMID: 31824312 PMC6880621

[236] Wang B Thompson KD Wangkahart E Yamkasem J Bondad-Reantaso MG Tattiyapong P. Strategies to enhance tilapia immunity to improve their health in aquaculture. Rev Aquaculture. (2023) 15:41–56. doi: 10.1111/raq.12731

[237] Lertwanakarn T Khemthong M Setthawong P Phaonakrop N Roytrakul S Ploypetch S. Proteomic and phosphoproteomic profilings reveal distinct cellular responses during Tilapinevirus tilapiae entry and replication. PeerJ. (2025) 13:e18923. doi: 10.7717/peerj.18923, PMID: 39995988 PMC11849505

[238] Kembou-Ringert JE Steinhagen D Thompson KD Daly JM Adamek M. Immune responses to Tilapia lake virus infection: what we know and what we don't know. Front Immunol. (2023) 14:1240094. doi: 10.3389/fimmu.2023.1240094, PMID: 37622112 PMC10445761

[239] Thanasaksiri K Hirono I Kondo H. Molecular cloning and expression analysis of NOD-like receptor 5 in Japanese flounder (Paralichthys olivaceus) after injection with two different formalin-killed pathogenic bacteria and poly (I:C). Dev Comp Immunol. (2017) 67:481–4. doi: 10.1016/j.dci.2016.08.017, PMID: 27592048

[240] Cardoso PHM Soares HS Martins ML Balian SC. Cryptocaryon irritans, a ciliate parasite of an ornamental reef fish yellowtail tang Zebrasoma xanthurum. Rev Bras Parasitol Vet. (2019) 28:750–3. doi: 10.1590/s1984-29612019033, PMID: 31215611

[241] Gurung P Kanneganti T-D. Immune responses against protozoan parasites: a focus on the emerging role of Nod-like receptors. Cell Mol Life Sci. (2016) 73:3035–51. doi: 10.1007/s00018-016-2212-3, PMID: 27032699 PMC4956549

[242] Finney CA Lu Z LeBourhis L Philpott DJ Kain KC. Disruption of Nod-like receptors alters inflammatory response to infection but does not confer protection in experimental cerebral malaria. Am J Trop Med hygiene. (2009) 80:718–22. doi: 10.4269/ajtmh.2009.80.718, PMID: 19407112

[243] Shaw MH Reimer T Sánchez-Valdepeñas C Warner N Kim Y-G Fresno M. T cell–intrinsic role of Nod2 in promoting type 1 immunity to Toxoplasma gondii. Nat Immunol. (2009) 10:1267–74. doi: 10.1038/ni.1816, PMID: 19881508 PMC2803073

[244] Santos MLS Reis EC Bricher PN Sousa TN Brito CFA Lacerda MVG. Contribution of inflammasome genetics in Plasmodium vivax malaria. Infect Genet Evol. (2016) 40:162–6. doi: 10.1016/j.meegid.2016.02.038, PMID: 26946405

[245] Shio MT Christian JG Jung JY Chang K-P Olivier M. PKC/ROS-mediated NLRP3 inflammasome activation is attenuated by leishmania zinc-metalloprotease during infection. PloS Negl Trop Dis. (2015) 9:e0003868. doi: 10.1371/journal.pntd.0003868, PMID: 26114647 PMC4482689

[246] Gurung P Karki R Vogel P Watanabe M Bix M Lamkanfi M. An NLRP3 inflammasome-triggered Th2-biased adaptive immune response promotes leishmaniasis. J Clin Invest. (2015) 125:1329–38. doi: 10.1172/JCI79526, PMID: 25689249 PMC4362229

[247] Lima-Junior DS Costa DL Carregaro V Cunha LD Silva ALN Mineo TWP. Inflammasome-derived IL-1β production induces nitric oxide–mediated resistance to Leishmania. Nat Med. (2013) 19:909–15. doi: 10.1038/nm.3221, PMID: 23749230

[248] Dostert C Guarda G Romero JF Menu P Gross O Tardivel A. Malarial hemozoin is a nalp3 inflammasome activating danger signal. PloS One. (2009) 4:e6510. doi: 10.1371/journal.pone.0006510, PMID: 19652710 PMC2714977

[249] Griffith JW Sun T McIntosh MT Bucala R. Pure hemozoin is inflammatory *in vivo* and activates the NALP3 inflammasome via release of uric acid1. J Immunol. (2009) 183:5208–20. doi: 10.4049/jimmunol.0713552, PMID: 19783673 PMC3612522

[250] Tiemi Shio M Eisenbarth SC Savaria M Vinet AF Bellemare M-J Harder KW. Malarial hemozoin activates the NLRP3 inflammasome through lyn and syk kinases. PloS Pathog. (2009) 5:e1000559. doi: 10.1371/annotation/abca067d-b82b-4de6-93c5-0fcc38e3df05, PMID: 19696895 PMC2722371

[251] Witola William H Mui E Hargrave A Liu S Hypolite M Montpetit A. NALP1 influences susceptibility to human congenital toxoplasmosis, proinflammatory cytokine response, and fate of toxoplasma gondii-infected monocytic cells. Infection Immun. (2011) 79:756–66. doi: 10.1128/IAI.00898-10, PMID: 21098108 PMC3028851

[252] Ewald Sarah E Chavarria-Smith J Boothroyd John C. NLRP1 is an inflammasome sensor for toxoplasma gondii. Infection Immun. (2014) 82:460–8. doi: 10.1128/IAI.01170-13, PMID: 24218483 PMC3911858

[253] Gov L Karimzadeh A Ueno N Lodoen Melissa B. Human innate immunity to toxoplasma gondii is mediated by host caspase-1 and ASC and parasite GRA15. mBio. (2013) 4:10. doi: 10.1128/mBio.00255-13, PMID: 23839215 PMC3705447

[254] Silva GK Gutierrez FRS Guedes PMM Horta CV Cunha LD Mineo TWP. Cutting Edge: Nucleotide-Binding Oligomerization Domain 1-Dependent Responses Account for Murine Resistance against Trypanosoma cruzi Infection. J Immunol. (2009) 184:1148–52. doi: 10.4049/jimmunol.0902254, PMID: 20042586

[255] Gonçalves VM Matteucci KC Buzzo CL Miollo BH Ferrante D Torrecilhas AC. NLRP3 Controls Trypanosoma cruzi Infection through a Caspase-1-Dependent IL-1R-Independent NO Production. PloS Negl Trop Dis. (2013) 7:e2469. doi: 10.1371/journal.pntd.0002469, PMID: 24098823 PMC3789781

[256] Silva GK Costa RS Silveira TN Caetano BC Horta CV Gutierrez FRS. Apoptosis-associated speck–like protein containing a caspase recruitment domain inflammasomes mediate IL-1β Response and host resistance to trypanosoma cruzi infection. J Immunol. (2013) 191:3373–83. doi: 10.4049/jimmunol.1203293, PMID: 23966627

[257] Ataide MA Andrade WA Zamboni DS Wang D M.d.C. Souza BS Elian S. Malaria-induced NLRP12/NLRP3-dependent caspase-1 activation mediates inflammation and hypersensitivity to bacterial superinfection. PloS Pathog. (2014) 10:e1003885. doi: 10.1371/journal.ppat.1003885, PMID: 24453977 PMC3894209

[258] Maisonneuve C Bertholet S Philpott DJ De Gregorio E. Unleashing the potential of NOD- and Toll-like agonists as vaccine adjuvants. Proc Natl Acad Sci U.S.A. (2014) 111:12294–9. doi: 10.1073/pnas.1400478111, PMID: 25136133 PMC4151741

[259] Ogawa C Liu YJ Kobayashi KS. Muramyl dipeptide and its derivatives: peptide adjuvant in immunological disorders and cancer therapy. Curr Bioact Compd. (2011) 7:180–97. doi: 10.2174/157340711796817913, PMID: 22180736 PMC3241611

[260] Behr MA Divangahi M. Freund's adjuvant, NOD2 and mycobacteria. Curr Opin Microbiol. (2015) 23:126–32. doi: 10.1016/j.mib.2014.11.015, PMID: 25483349

[261] Bahr GM. Non-specific immunotherapy of HIV-1 infection: potential use of the synthetic immunodulator murabutide. J Antimicrobial Chemotherapy. (2003) 51:5–8. doi: 10.1093/jac/dkg063, PMID: 12493780

[262] Darcissac ECA Truong M-J Dewulf J Mouton Y Capron A Bahr GM. The synthetic immunomodulator murabutide controls human immunodeficiency virus type 1 replication at multiple levels in macrophages and dendritic cells. J Virol. (2000) 74:7794–802. doi: 10.1128/JVI.74.17.7794-7802.2000, PMID: 10933686 PMC112309

[263] Hancock RE Nijnik A Philpott DJ. Modulating immunity as a therapy for bacterial infections. Nat Rev Microbiol. (2012) 10:243–54. doi: 10.1038/nrmicro2745, PMID: 22421877

[264] Fritz JH Le Bourhis L Sellge G Magalhaes JG Fsihi H Kufer TA. Nod1-mediated innate immune recognition of peptidoglycan contributes to the onset of adaptive immunity. Immunity. (2007) 26:445–59. doi: 10.1016/j.immuni.2007.03.009, PMID: 17433730

[265] Bourhis LL. Nod1 and Nod2 in innate immune responses, adaptive immunity and bacterial infection. Departmenent Immunology Univ Toronto. (2009) p:160. Available online at: http://hdl.handle.net/1807/24313.

[266] O’Hagan DT De Gregorio E. The path to a successful vaccine adjuvant – ‘The long and winding road’. Drug Discov Today. (2009) 14:541–51. doi: 10.1016/j.drudis.2009.02.009, PMID: 19508916

[267] Tada H Aiba S Shibata K Ohteki T Takada H. Synergistic effect of Nod1 and Nod2 agonists with toll-like receptor agonists on human dendritic cells to generate interleukin-12 and T helper type 1 cells. Infect Immun. (2005) 73:7967–76. doi: 10.1128/IAI.73.12.7967-7976.2005, PMID: 16299289 PMC1307098

[268] Pavot V Rochereau N Primard C Genin C Perouzel E Lioux T. Encapsulation of Nod1 and Nod2 receptor ligands into poly(lactic acid) nanoparticles potentiates their immune properties. J Control Release. (2013) 167:60–7. doi: 10.1016/j.jconrel.2013.01.015, PMID: 23352911

[269] He J Meng Z Lu D Liu X Lin H. Recognition of MDP and activation of NF-κB by cytosolic sensor NOD2 in Oreochromis niloticus. Aquaculture. (2021) 540:736700. doi: 10.1016/j.aquaculture.2021.736700, PMID: 33444736

[270] Lee KM Kim KH. Long-term and non-specific immune memory induced by muramyl dipeptide and its protective effect against Vibrio Anguillarum in rainbow trout (Oncorhynchus mykiss). Dev Comp Immunol. (2025) 167:105386. doi: 10.1016/j.dci.2025.105386, PMID: 40334803

[271] Philpott DJ Sorbara MT Robertson SJ Croitoru K Girardin SE. NOD proteins: regulators of inflammation in health and disease. Nat Rev Immunol. (2014) 14:9–23. doi: 10.1038/nri3565, PMID: 24336102

[272] Miryala KR Swain B. Advances and challenges in aeromonas hydrophila vaccine development: immunological insights and future perspectives. Vaccines. (2025) 13:202. doi: 10.3390/vaccines13020202, PMID: 40006748 PMC11861604

[273] Rathor GS Swain B. Advancements in fish vaccination: current innovations and future horizons in aquaculture health management. Appl Sci. (2024) 14:5672. doi: 10.3390/app14135672

