# Supplementary material for: NOD-like receptors in fish: evolution, structure, immune signaling, and targeting for aquaculture vaccine adjuvants
Source: Front Immunol. 2025 Sep 2;16:1665071. doi: 10.3389/fimmu.2025.1665071 (PMC12436146; doi:10.3389/fimmu.2025.1665071)
Supplement: Supplementary file 1 [file Table1.docx]

**Table S1: Accession numbers of organisms from the phylogenetic tree**

| Gene | Organism | | Accession number |
| --- | --- | --- | --- |
|  | **Scientific Name** | **Common name** |  |
| NOD1 | *Danio rerio* | Zebrafish | AGO14286.1 |
|  | *Ictalurus punctatus* | Channel catfish | NP_001186996.1 |
|  | *Carassius auratus* | Goldfish | AFY26968.1 |
|  | *Paralichthys olivaceus* | Olive flounder | AFD29895.1 |
|  | *Oreochromis niloticus* | Nile Tilapia | AVK93184.1 |
|  | *Labeo rohita* | Rohu | AFE61355.1 |
|  | *Ctenopharyngodon idella* | Grass Carp | ACX71752.1 |
|  | *Miichthys miiuy* | Miiuy croaker | AKR76245.1 |
|  | *Epinephelus coioides* | Orange-spotted grouper | AFV53357.1 |
|  | *Oncorhynchus mykiss* | Rainbow trout | AII73558.1 |
|  | *Takifugu rubripes* | Japanese pufferfish | XP_003965935.3 |
|  | *Channa punctata* | Spotted snakehead | QDH76353.1 |
|  | *megalobrama amblycephala* | Wuchang bream | XP_048008679.1 |
|  | *Danio aesculapii* | Panther danio | XP_056331088.1 |
|  | *Chanodichthys erythropterus* | Predatory carp | XP_067267321.1 |
|  | *Carassius gibelio* | Prussian carp | XP_052473745.1 |
|  | *Carassius carassius* | Crucian carp | XP_059364565.1 |
|  | *Pimephales promelas* | Fathead minnow | AYN78120.1 |
|  | *Colossoma macropomum* | Tambaqui | XP_036413994.1 |
|  | *Clarias magur* | Walking catfish | KAF5883742.1 |
|  | *Silurus asotus* | Amur catfish | KAI5615176.1 |
|  | *Silurus meridionalis* | Yangtze catfish | KAI5086765.1 |
|  | *Clupea harengus* | Atlantic herring | XP_012694256.1 |
|  | *Oncorhynchus nerka* | Sockeye salmon | XP_029478259.2 |
|  | *Mus musculus* | Mouse | NP_001164478.1 |
|  | *Dendropsophus ebraccatus* | Hourglass tree frog | XP_069816386.1 |
|  | *Homo sapiens* | Human | AAD28350.1 |
|  | *Gallus gallus domesticus* | Chicken | NP_001305367.1 |
|  | *Alligator mississippiensis* | American alligator | XP_014458734.1 |
| NOD2 | *Danio rerio* | Zebrafish | NP_001314973.1 |
|  | *Ictalurus punctatus* | Channel catfish | NP_001186996.1 |
|  | *Carassius auratus* | Goldfish | AFY26969.1 |
|  | *Oreochromis niloticus* | Nile Tilapia | AUN88447.1 |
|  | *Labeo rohita* | Rohu | XP_050970448.1 |
|  | *Ctenopharyngodon idella* | Grass Carp | ACX71753.1 |
|  | *Miichthys miiuy* | Miiuy croaker | AKR76246.1 |
|  | *Epinephelus coioides* | Orange-spotted grouper | AFV53358.1 |
|  | *Oncorhynchus mykiss* | Rainbow trout | NP_001188484.1 |
|  | *Takifugu rubripes* | Japanese pufferfish | NP_001035913.1 |
|  | *Colossoma macropomum* | Tambaqui | XP_036440399.1 |
|  | *Oncorhynchus gorbuscha* | Pink salmon | XP_046200978.1 |
|  | *Coregonus clupeaformis* | Lake whitefish | XP_045073450.1 |
|  | *Micropterus dolomieu* | Smallmouth bass | XP_045887719.1 |
|  | *Centropristis striata* | Black sea bass | XP_059206540.1 |
|  | *Perca flavescens* | Yellow perch | XP_028442713.1 |
|  | *Semicossyphus pulcher* | California sheephead | XP_069513561.1 |
|  | *Amphiprion ocellaris* | Clownfish | XP_023131605.2 |
|  | *Perca fluviatilis* | European perch | XP_039651998.1 |
|  | *Dicentrarchus labrax* | European seabass | XP_051236173.1 |
|  | *Morone saxatilis* | Striped bass | XP_035537984.1 |
|  | *Seriola lalandi* | Yellowtail amberjack | WEG20044.1 |
|  | *Xiphias gladius* | Swordfish | XP_039993838.1 |
|  | *Thunnus albacares* | Yellowfin tuna | XP_044216328.1 |
|  | *Seriola dumerili* | Greater amberjack | XP_022597963.1 |
|  | *Thunnus maccoyii* | Southern bluefin tuna | XP_042268373.1 |
|  | *Thunnus thynnus* | Atlantic bluefin tuna | XP_067451149.1 |
|  | *Trachinotus ovatus* | Pompano | URH23959.1 |
|  | *Pelmatolapia mariae* | Spotted tilapia | XP_063323085.1 |
|  | *Micropterus salmoides* | Largemouth bass | XP_038556990.1 |
|  | *Scomber scombrus* | Atlantic mackerel | XP_062297747.1 |
|  | *Mus musculus* | Mouse | AAN84594.1 |
|  | *Dendropsophus ebraccatus* | Hourglass tree frog | XP_069822038.1 |
|  | *Homo sapiens* | Human | AAG33677.1 |
| NLRC3 | *Danio rerio* | Zebrafish | XP_009295904.1 |
|  | *Ictalurus punctatus* | Channel Catfish | XP_017326803.1 |
|  | *Carassius auratus* | Goldfish | XP_026056452.1 |
|  | *Oreochromis niloticus* | Nile Tilapia | AUN88448.1 |
|  | *Labeo rohita* | Rohu | XP_050954269.1 |
|  | *Ctenopharyngodon idella* | Grass Carp | XP_051737605.1 |
|  | *Miichthys miiuy* | Miiuy croaker | ALJ32257.1 |
|  | *Oncorhynchus mykiss* | Rainbow trout | APD13816.1 |
|  | *Scophthalmus maximus* | Turbot | QHQ71236.1 |
|  | *Salmo salar* | Atlantic salmon | XP_045558500.1 |
|  | *Danio aesculapii* | Panther danio | XP_056306830.1 |
|  | *Carassius gibelio* | Prussian carp | XP_052451976.1 |
|  | *Carassius carassius* | Crucian carp | XP_059381114.1 |
|  | *Colossoma macropomum* | Tambaqui | XP_036447053.1 |
|  | *Silurus asotus* | Amur catfish | KAI5624163.1 |
|  | *Silurus meridionalis* | Yangtze catfish | XP_046702956.1 |
|  | *Oncorhynchus gorbuscha* | Pink salmon | XP_046225018.1 |
|  | *Silurus meridionalis* | Yangtze catfish | XP_046702956.1 |
|  | *Tachysurus fulvidraco* | Yellowhead catfish | XP_027016817.2 |
|  | *Ictalurus furcatus* | Blue catfish | XP_053476304.1 |
|  | *Oncorhynchus kisutch* | Coho salmon | XP_020309317.1 |
|  | *Salmo trutta* | Brown trout | XP_029565648.1 |
|  | *Mus musculus* | Mouse | Q5DU56.2 |
|  | *Dendropsophus ebraccatus* | Hourglass tree frog | XP_069840208.1 |
|  | *Homo sapiens* | Human | ACP40993.1 |
|  | *Gallus gallus domesticus* | Chicken | QWT69015.1 |
|  | *Alligator mississippiensis* | American alligator | KYO32396.1 |
| NLRC5 | *Danio rerio* | Zebrafish | AFN73230.1 |
|  | *Ictalurus punctatus* | Channel catfish | NP_001186995.1 |
|  | *Carassius auratus* | Goldfish | XP_026143196.1 |
|  | *Paralichthys olivaceus* | Olive flounder | BAW27607.1 |
|  | *Labeo rohita* | Rohu | XP_050991204.1 |
|  | *Miichthys miiuy* | Miiuy croaker | ALJ32258.1 |
|  | *Oncorhynchus mykiss* | Rainbow trout | APD13817.1 |
|  | *Salmo salar* | Atlantic salmon | XP_014004499.1 |
|  | *Chanodichthys erythropterus* | Predatory carp | XP_067246383.1 |
|  | *Carassius gibelio* | Prussian carp | XP_052438933.1 |
|  | *Carassius carassius* | Crucian carp | XP_059362578.1 |
|  | *Colossoma macropomum* | Tambaqui | XP_036441247.1 |
|  | *Tachysurus fulvidraco* | Yellowhead catfish | XP_027017528.2 |
|  | *Mus musculus* | Mouse | C3VPR6.2 |
|  | *Dendropsophus ebraccatus* | Hourglass tree frog | XP_069822103.1 |
|  | *Homo sapiens* | Human | NP_115582.4 |
|  | *Gallus gallus domesticus* | Chicken | AEY11256.1 |
|  | *Alligator mississippiensis* | American alligator | XP_059569277.1 |
| NLRX1 | *Danio rerio* | Zebrafish | WET20154.1 |
|  | *Ictalurus punctatus* | Channel catfish | NP_001186993.1 |
|  | *Carassius auratus* | Goldfish | AFY26970.1 |
|  | *Labeo rohita* | Rohu | XP_050985834.1 |
|  | *Ctenopharyngodon idella* | Grass Carp | AXK69168.1 |
|  | *Miichthys miiuy* | Miiuy croaker | ALJ32259.1 |
|  | *Oncorhynchus mykiss* | Rainbow trout | APD13818.1 |
|  | *Takifugu rubripes* | Japanese pufferfish | XP_011616357.1 |
|  | *Channa punctata* | Spotted snakehead | QDH76354.1 |
|  | *Scophthalmus maximus* | Turbot | QHQ71238.1 |
|  | *Micropterus dolomieu* | Smallmouth bass | XP_045930013.1 |
|  | *Centropristis striata* | Black sea bass | XP_059187224.1 |
|  | *Perca flavescens* | Yellow perch | XP_028430150.1 |
|  | *Perca fluviatilis* | European perch | XP_039643122.1 |
|  | *Morone saxatilis* | Striped bass | XP_035535427.1 |
|  | *Seriola dumerili* | Greater amberjack | XP_022619206.1 |
|  | *Takifugu flavidus* | Yellowbelly pufferfish | XP_056906585.1 |
|  | *Mus musculus* | Mice | NP_001157215.1 |
|  | *Dendropsophus ebraccatus* | Hourglass tree frog | XP_069803241.1 |
|  | *Homo sapiens* | Human | AAI10891.1 |
|  | *Gallus gallus domesticus* | Chicken | XP_040546147.1 |
|  | *Alligator mississippiensis* | American alligator | XP_019345737.2 |
| NLRP1 | *Danio rerio* | Zebrafish | AYC80945.1 |
|  | *Labeo rohita* | Rohu | XP_050992902.1 |
|  | *Cyprinus carpio* | Common carp | UPG20634.1 |
|  | *Megalobrama amblycephala* | Wuchang bream | XP_048020090.1 |
|  | *Danio aesculapii* | Panther danio | XP_056336858.1 |
|  | *Chanodichthys erythropterus* | Predatory carp | XP_067219292.1 |
|  | *Carassius carassius* | Crucian carp | XP_059358628.1 |
|  | *Mus musculus* | Mouse | NP_001004142.2 |
|  | *Homo sapiens* | Human | AAH51787.1 |
